# Comparative effectiveness of antimicrobial regimens for pneumonia caused by drug-resistant *Acinetobacter baumannii*: a network meta-analysis including cefiderocol and inhaled therapies

**DOI:** 10.1186/s12879-026-12640-z

**Published:** 2026-01-20

**Authors:** Ming-Ying Ai, Wei-Lun Chang

**Affiliations:** Department of Pharmacy, Far Eastern Memorial Hospital, New Taipei City, Taiwan

**Keywords:** Cefiderocol-containing regimes, Colistin, Multidrug-resistant *Acinetobacter baumannii*, Nosocomial pneumonia, Mortality

## Abstract

**Background:**

The objective of this network meta-analysis was to evaluate and compare the efficacy and safety of different antimicrobial regimens used in the treatment of pneumonia caused by extensively drug-resistant (XDR) or multidrug-resistant (MDR) *Acinetobacter baumannii* (AB). Given the increasing prevalence of resistant strains, identifying optimal treatment strategies is crucial.

**Materials and methods:**

We systematically analyzed data from randomized controlled trials and retrospective cohort studies retrieved from major electronic databases. The included studies evaluated all-cause mortality, clinical success, microbiological eradication, and nephrotoxicity associated with cefiderocol, intravenous (IV) colistin, inhaled colistin, igecycline, sulbactam, and their combination-based regimens in patients with MDR/XDR-AB pneumonia.

**Results:**

A total of 19 eligible studies involving 1,941 participants were included in the analysis. Cefiderocol-containing regimens demonstrated the greatest reduction in all-cause mortality (odds ratio (OR): 0.24; 95% CI: 0.09–0.68) compared to other therapies. In terms of clinical success, cefiderocol-containing regimens (OR: 2.77; 95% CI: 1.07–7.19) and inhaled colistin (OR: 2.61; 95% CI: 1.14–5.99) were significantly more effective than other regimens. Microbiological eradication was most notable with inhaled colistin, and with IV colistin combined with sulbactam or tigecycline. However, nephrotoxicity was commonly observed with IV colistin, while tigecycline monotherapy showed the lowest nephrotoxicity risk (OR: 0.15; 95% CI: 0.04–0.60).

**Conclusion:**

This study provides comprehensive evidence on current and emerging treatments for MDR/XDR-AB pneumonia. Cefiderocol-containing regimens appears to be the most effective regimen in reducing mortality and improving clinical outcomes, though nephrotoxicity risks must be carefully considered when using IV colistin-based therapies.

**Clinical trial:**

Not applicable.

**Supplementary Information:**

The online version contains supplementary material available at 10.1186/s12879-026-12640-z.

## Introduction

*Acinetobacter baumannii* (AB) has emerged as a formidable nosocomial pathogen, particularly affecting critically ill and immunocompromised individuals. Pneumonia caused by multidrug-resistant (MDR) and extensively drug-resistant (XDR) strains especially ventilator-associated pneumonia (VAP) represents a significant global health concern [1]. This threat is primarily attributed to the organism’s exceptional ability to persist in harsh hospital environments, swiftly acquire antimicrobial resistance, and form robust biofilms on medical devices. Consequently, AB pneumonia has become one of the leading causes of healthcare-associated infections, particularly within intensive care units (ICUs) around the world [2].

AB is recognized as one of the ESKAPE pathogens a prominent group of MDR organisms known for their ability to “escape” the effects of antimicrobial agents and cause severe healthcare associated infections. The ESKAPE group, comprising *Enterococcus faecium*, *Staphylococcus aureus*, *Klebsiella pneumoniae*, *Acinetobacter baumannii*, *Pseudomonas aeruginosa*, and *Enterobacter* species, was first conceptualized to highlight pathogens of highest clinical concern due to their virulence, adaptability, and extensive drug resistance [3]. Subsequent global surveillance studies and the World Health Organization (WHO) have consistently designated AB, particularly carbapenem-resistant strains, as a critical priority pathogen requiring urgent research and development of new therapeutic options. As one of the most challenging members of the ESKAPE group, AB is associated with high mortality in VAP and bloodstream infections (BSI), driven by its exceptional environmental persistence, biofilm formation, and rapid acquisition of resistance determinants. Mechanisms of resistance include the production of carbapenemases (notably OXA-type β-lactamases), overexpression of efflux pumps, alterations in outer membrane porins, and target site mutations. These multifaceted resistance strategies enable AB to evade most conventional antibiotics, making infections difficult to treat and often resulting in poor clinical outcomes [4].

Although the epidemiology of AB infections varies geographically, global surveillance data consistently highlight their substantial clinical and public health burden. In many regions across Asia, Southern Europe, the Middle East, and Latin America, carbapenem-resistant and XDR-AB account for 30–70% of *Acinetobacter* isolates, representing some of the highest antimicrobial resistance rates worldwide [5]. These strains are strongly associated with severe hospital acquired infections particularly VAP with mortality rates frequently exceeding 40–60% in critically ill populations [6]. Even in regions with lower prevalence, AB remains a persistent cause of healthcare associated outbreaks due to its remarkable environmental resilience. Incorporating these global and regional epidemiologic patterns underscores the urgent need to evaluate effective therapeutic strategies for MDR/XDR-AB treatment.

The current therapeutic arsenal against MDR/XDR AB is extremely limited, and treatment recommendations rely heavily on contemporary guideline statements from Infectious Diseases Society of America (IDSA) and European Society of Clinical Microbiology and Infectious Diseases (ESCMID), which emphasize individualized regimen selection based on disease severity and antimicrobial susceptibility [7, 8]. Traditionally used antibiotics such as carbapenems and aminoglycosides have largely lost effectiveness owing to widespread resistance. As a result, management has depended on several “last-resort” agents polymyxins (colistin or polymyxin B), tigecycline, high-dose sulbactam, and more recently cefiderocol, each with notable limitations. Colistin, although frequently used, is associated with dose limiting nephrotoxicity and poor pulmonary penetration, raising concerns regarding its reliability for pneumonia. Tigecycline also demonstrates low serum and epithelial lining fluid concentrations, limiting its effectiveness in severe pulmonary infections. High-dose sulbactam, which possesses unique intrinsic activity against AB, shows promise and is recommended by guidelines as part of combination therapy for carbapenem resistant *Acinetobacter baumannii* (CRAB); however, the supporting evidence remains limited to small observational studies [9].

The optimal treatment approach monotherapy versus combination therapy remains an area of ongoing debate. Monotherapy with an active agent may suffice in selected cases with less severe disease, but the evidence generally supports the use of combination regimens in patients with critical illness or infections caused by XDR strains. Combinations often aim to achieve synergistic effects, reduce resistance emergence, and improve clinical outcomes. Common combinations include colistin with high dose sulbactam, tigecycline, carbapenems or rifampin [10]. Nevertheless, the clinical evidence supporting these regimens is largely derived from observational studies and small retrospective cohorts, and robust randomized controlled trials are lacking. In addition, the rising incidence of resistance even to colistin previously considered a reliable last line agent further complicates treatment choices.

Cefiderocol, a novel siderophore cephalosporin, has demonstrated potent in vitro activity against MDR- and XDR-AB, offering a promising therapeutic option for difficult to treat pneumonia [11]. While early clinical trials and observational studies suggest potential efficacy, real world outcomes remain inconsistent, particularly in critically ill patients. Concerns have been raised regarding resistance development during therapy and suboptimal clinical response despite favorable susceptibility profiles [12]. Additionally, limited data from randomized controlled trials specifically targeting AB pneumonia hinder definitive conclusions.

Given the rising prevalence of MDR- and XDR-AB pneumonia and the limited effectiveness of currently available treatment options, identifying optimal therapeutic strategies has become an urgent clinical priority. Although agents like colistin, tigecycline, and high dose sulbactam have been used, their efficacy remains suboptimal and often inconsistent. The introduction of cefiderocol has sparked interest as a potential alternative; however, real world evidence remains limited, and its role within combination regimens is not yet well defined. Therefore, this study aims to evaluate and compare the effectiveness of different antimicrobial regimens for the treatment of MDR/XDR-AB pneumonia, with a focus on clinical outcomes and mortality.

## Materials and methods

This study was conducted according to the Preferred Reporting Items for Systematic Reviews and Meta-Analysis (PRISMA) extension guidelines specific to network meta-analysis (PRISMA-NMA) [13]. It has been registered on the International Platform of Registered Systematic Review and Meta-analysis Protocols (INPLASY) under registration number INPLASY2025-8-0032 [14]. Ethical approval and informed consent were not required for this research.

### Database searches and study identification

Two authors (M-YA and W-LC) independently performed electronic database searches using PubMed, Cochrane Reviews, Cochrane Central, Web of Science, and ClinicalTrials.gov. The search was conducted using the keywords: (‘*Acinetobacter baumannii*’) AND (‘Pneumonia’) AND (‘mortality’). The literature search for the systematic review and network meta-analysis covered all available studies from database inception to the most recent search date (April 1, 2025). Initially, both authors independently screened titles and abstracts to evaluate study eligibility, resolving disagreements through consensus discussion. If consensus was unattainable, a third reviewer was consulted for resolution. No language restrictions were applied during the literature search.

### Inclusion and exclusion criteria

The network meta-analysis was structured based on the PICO (population, intervention, comparison, and outcome) framework, which included the following criteria: (1) Population (P): human patients with MDR/XRD AB VAP or Hospital-acquired pneumonia (HAP) treated with mono- or combination therapy antibiotics. (2) Intervention (I): Patients received either monotherapy or combination antibiotic regimens that included at least one of the following agents: intravenous (IV) or inhaled (IH) colistin, polymyxin B, tigecycline, sulbactam, ampicillin-sulbactam, sulbactam-durlobactam, or cefiderocol. These agents are currently recommended for their demonstrated efficacy against MDR- and XDR-AB. (3) Comparison (C): IV colistin; and (4) Outcome (O): all caused mortality, clinical success, microbiological eradication and risk of nephrotoxicity.

The inclusion criteria for eligible studies were as follows: (1) randomized controlled trials (RCTs), retrospective or prospective study involving patients with MDR/XRD-AB VAP or HAP undergoing antibiotics treatment; (2) studies with follow up durations longer than two weeks; (3) Studies were included if they explicitly evaluated at least one of the following outcomes: all-cause mortality, clinical success, or microbiological eradication. The primary outcome selected for assessing antimicrobial effectiveness was all-cause mortality, defined as death from any cause occurring within approximately 30 days. The other outcomes were clinical success, microbiological eradication and nephrotoxicity. Clinical success was defined as the complete resolution of pneumonia associated clinical signs and symptoms by the end of treatment. Microbiological eradication was defined as a confirmed absence of bacteria in follow up cultures obtained at the completion of antibiotic therapy.

The exclusion criteria for our systematic review and network meta-analysis were as follows: (1) case reports; (2) studies involving pediatric populations; (3) patients diagnosed with community-acquired pneumonia; (4) patients with infections at sites other than the lung; (5) patients diagnosed with pneumonia caused by other Gram-positive cocci (GPC) or Gram-negative bacilli (GNB) contemporary; (6) studies with incomplete or inaccessible data despite attempts to contact authors via email; and (7) studies with overlapping patient populations already included in the analysis.

### Modeling for network meta-analysis

In conducting this network meta-analysis, we adhered to specific guidelines to ensure consistency and minimize variability during the construction of our analytical model. Paired comparisons were strictly limited to antibiotic regimens versus IV colistin for treating pneumonia. Studies involving infections other than HAP or VAP were intentionally excluded, as inclusion of diverse infection sites could introduce substantial heterogeneity into the analysis due to differing pharmacological characteristics, potentially leading to inconsistent results [15].

### Methodological quality appraisal

To evaluate the methodological quality of the studies included in our analysis, we assessed non-randomized studies using the Newcastle-Ottawa Scale (NOS) [16]. The NOS evaluates study quality across three domains: the selection of study participants, comparability between groups, and the ascertainment of outcomes. For randomized controlled trials, methodological rigor was evaluated using the revised Cochrane Risk of Bias tool (RoB 2), which assesses potential bias across domains such as the randomization process, deviations from intended interventions, missing outcome data, outcome measurement, and selective reporting.

### Primary outcome

The primary outcome of this study was all-cause mortality among patients with MDR/XDR AB pneumonia treated with different antimicrobial regimens. Each regimen was defined as monotherapy or combination therapy using antibiotics with documented activity against MDR/XDR AB. Mortality was extracted as reported in each study and served as the principal comparative measure across treatment arms.

### Secondary outcomes

The secondary outcomes included clinical success and microbiological eradication, evaluated according to the definitions provided in the original studies. In addition, we assessed nephrotoxicity, comparing the odds of renal toxicity among different antimicrobial regimens, with intravenous colistin monotherapy used as the reference group. The secondary analyses were conducted to provide a broader understanding of treatment effectiveness and safety.

### Data extraction, management, and conversion

Data extraction was independently performed by two authors (M-YA and W-LC). This process entailed collecting a range of information from the included studies, including demographic characteristics, study design, details of the interventions, and both primary and secondary outcomes. The procedures for data extraction, transformation, and synthesis were carried out in accordance with the guidelines outlined in the Cochrane Handbook for Systematic Reviews of Interventions, as well as other relevant medical literature [17–21].

### Statistical analyses

Due to the heterogeneity among the included studies, a random effects model was used for the network meta-analysis. The analyses were conducted using MetaInsight software (version 4.0.2, Complex Reviews Support Unit, National Institute for Health Research, London, UK) within a frequentist statistical framework. MetaInsight, a web-based tool for network meta-analysis, employs the netmeta package in R software for frequentist statistical analyses. In this model, the restricted maximum likelihood (REML) estimator was used to estimate between study variance (τ²).

Initially, forest plots and network plots were generated to visually depict pairwise comparisons among individual studies. Additional forest plots were created to evaluate the odds ratios for all-cause mortality, clinical success, microbiological eradication, and renal toxicity compared with the intravenous colistin group. These plots illustrated the outcomes by presenting odds ratio as point estimates accompanied by 95% confidence intervals (95% CI). Treatment ranking within the frequentist framework was derived using P-scores, which estimate the relative probability that each regimen is superior to competing treatments. Numerical results for both direct and indirect comparisons were summarized in tables. Inconsistency was assessed using a local node-splitting approach, which compares direct and indirect evidence for each pairwise comparison. A two tailed *p*-value of less than 0.05 was considered statistically significant.

### Sensitivity analysis

To enhance the reliability of our study results, we performed a one study removed sensitivity analysis. This method involved sequentially excluding each study to ensure that no single study disproportionately influenced the overall findings. By systematically removing each study from the analyses of all-cause mortality, clinical success, microbiological eradication, and renal toxicity, we assessed the stability of our conclusions and the rankings of the studies.

### Publication bias

We assessed potential publication bias according to the guidelines outlined in the Cochrane Handbook for Systematic Reviews of Interventions [17]. To visualize potential bias, we constructed funnel plots using Comprehensive Meta-Analysis software, version 4 (BioStat, Englewood, NJ, USA), focusing specifically on comparisons with the intravenous colistin group. Additionally, Egger’s regression test was employed to quantitatively evaluate the extent and significance of any publication bias.

**Fig. 1:**
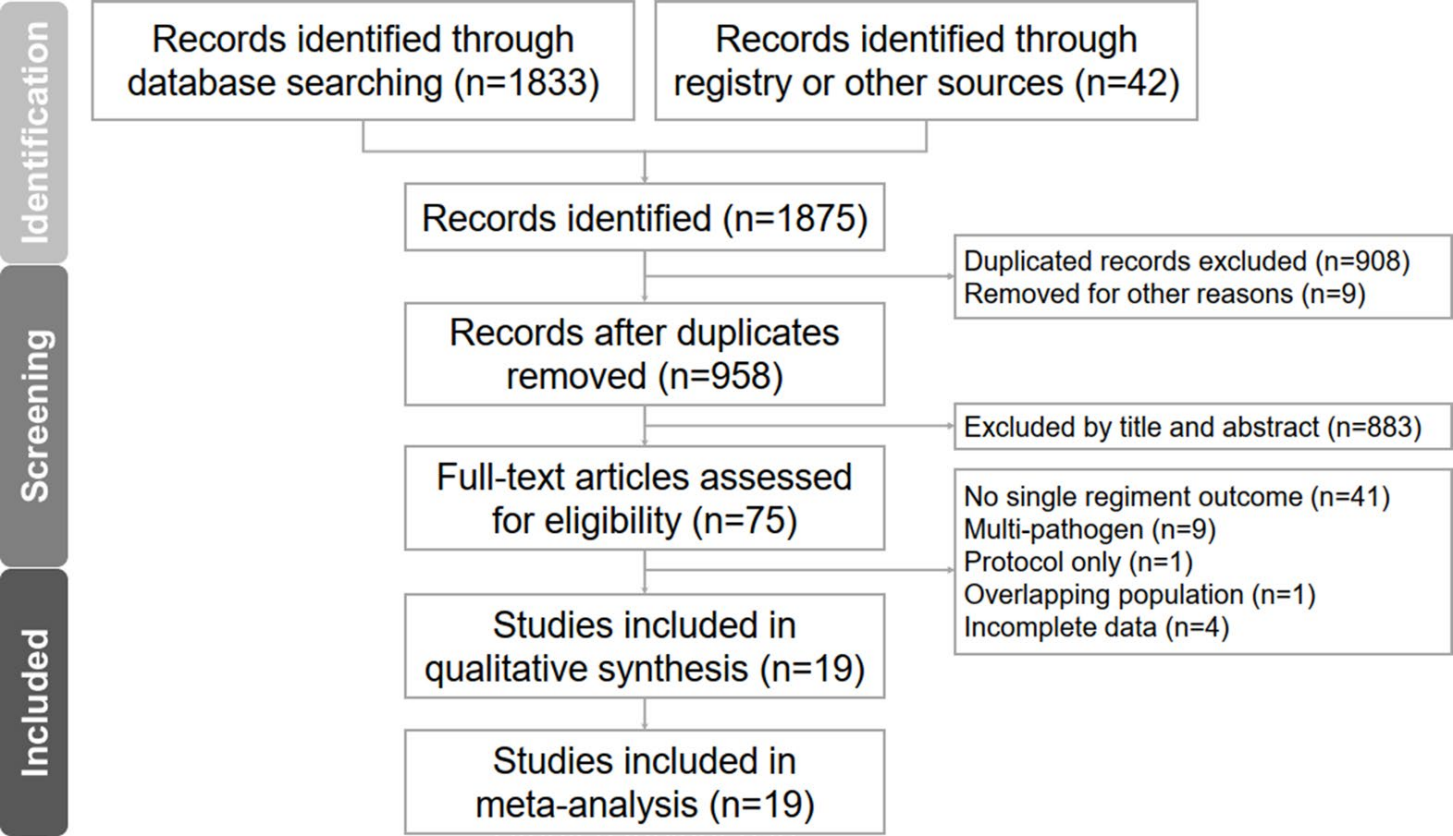
PRISMA flowchart

## Results

### Study identification and network model formation

Figure 1 presents the PRISMA flowchart outlining the process of our literature search.

The PRISMA NMA extension checklist is provided in Supplementary Table S1. The selection process involved removing duplicate articles and excluding those unrelated to our research based on title and abstract screening, ultimately resulting in the inclusion of 19 studies [22–40]. These studies comprised randomized controlled trials as well as retrospective and prospective cohort studies.

**Fig. 2:**
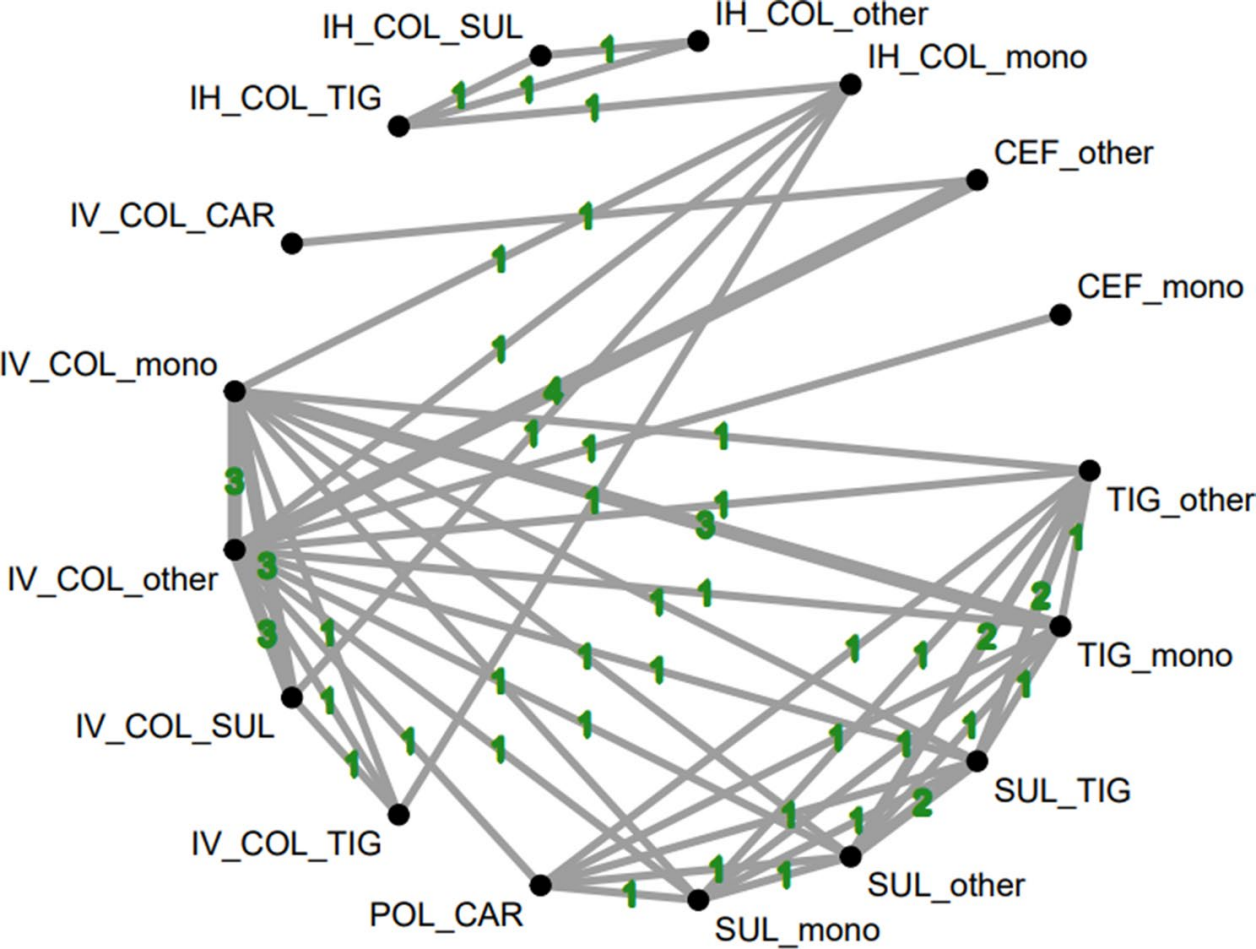
The network plots demonstrate the impact of various regimens for MDR/XDR *Acinetobacter baumannii* pneumonia on all-cause mortality, with the number of contributing trials indicated on each line

In total, our analysis included 19 studies encompassing 1,941 participants. The regimens evaluated across these studies included the following treatment nodes observed in the network: cefiderocol monotherapy (CEF mono), cefiderocol plus other agents (CEF + other), inhaled colistin monotherapy (IH COL mono), inhaled colistin plus other agents (IH COL + other), inhaled colistin plus sulbactam (IH COL + SUL), inhaled colistin plus tigecycline (IH COL + TIG), intravenous colistin monotherapy (IV COL mono), intravenous colistin plus carbapenem (IV COL + CAR), intravenous colistin plus other agents (IV COL + other), intravenous colistin plus sulbactam (IV COL + SUL), intravenous colistin plus tigecycline (IV COL + TIG), polymyxin B plus carbapenem (POL + CAR), sulbactam monotherapy (SUL mono), sulbactam plus other agents (SUL + other), sulbactam plus tigecycline (SUL + TIG), tigecycline monotherapy (TIG mono), and tigecycline plus other agents (TIG + other). Figure 2 illustrates the network model representing each regimen. Further details regarding the inclusion criteria and characteristics of the included studies are provided in Table 1.

### Methodological quality of the included studies

The assessment of methodological quality for the included studies is presented in Supplementary Table S2. Studies identified as having a potential risk of bias exhibited protocol variations among different study groups, which may have affected adherence and the outcomes of the interventions.

### Primary outcome: all-cause mortality

**Fig. 3:**
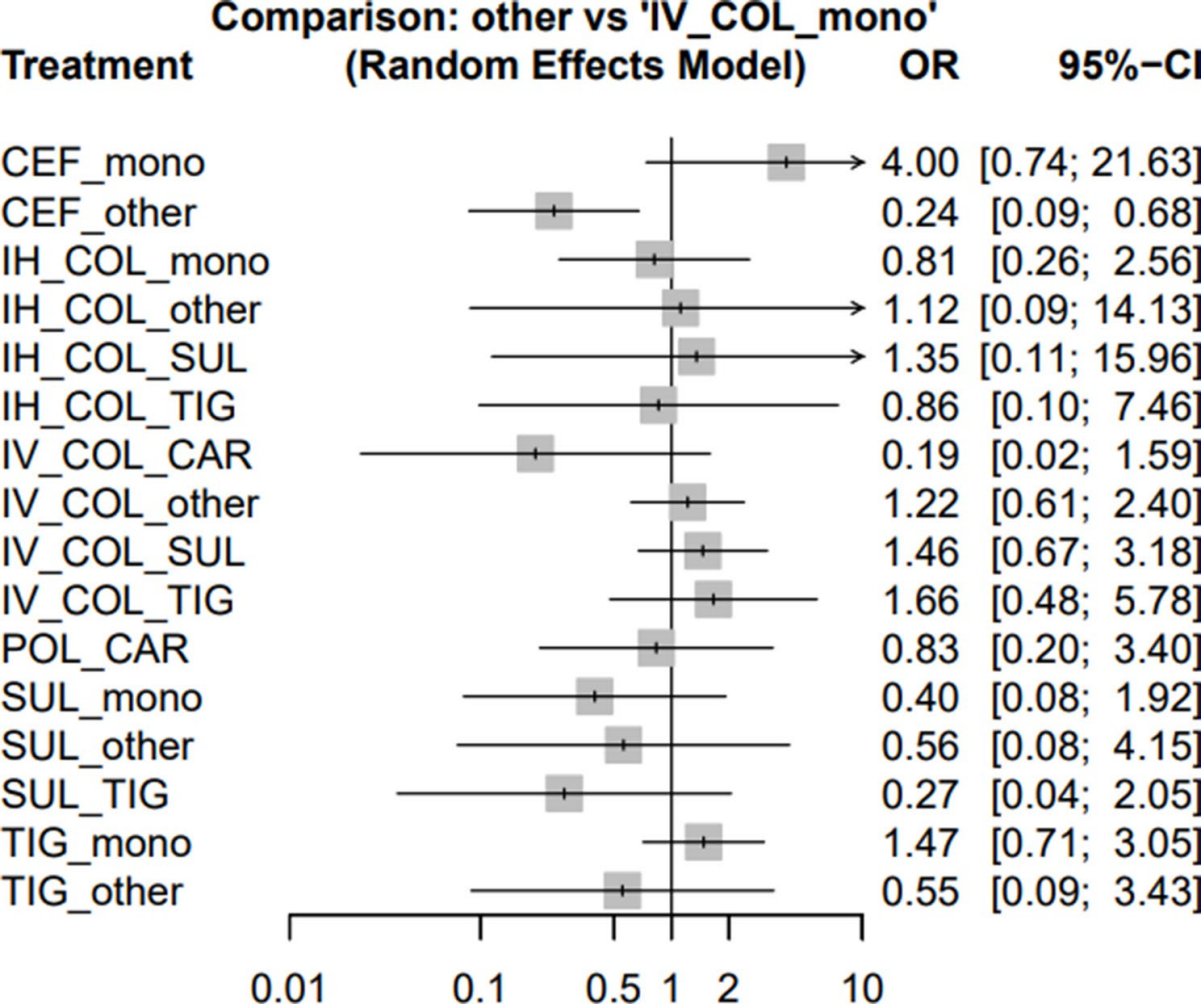
Forest plots illustrating the odds ratios (ORs) for all-cause mortality associated with different regimens used to treat MDR/XDR *Acinetobacter baumannii* pneumonia

Across the comparative analyses using IV colistin monotherapy as the reference, several regimens demonstrated a trend toward reduced all-cause mortality. Combination therapies involving cefiderocol, tigecycline, or sulbactam generally showed lower odds of death compared with IV colistin alone, although most estimates did not reach statistical significance. Notably, cefiderocol + other agent exhibited a significantly protective effect (OR 0.24; 95% CI 0.09–0.68), suggesting potential benefit when cefiderocol was combined with another agent. Regimens such as sulbactam + tigecycline (OR 0.27; 95% CI 0.04–2.05), tigecycline + other agent (OR 0.55; 95% CI 0.09–3.43), and sulbactam + other agent (OR 0.56; 95% CI 0.08–4.15) also favored lower mortality, though confidence intervals remained wide. Conversely, cefiderocol monotherapy showed a numerically higher mortality risk (OR 4.00; 95% CI 0.74–21.63), likely driven by small sample sizes. Most inhaled colistin containing regimens demonstrated neutral effects (Fig. 3).

**Table 1:** Summary of included studies reporting clinical outcomes including all-cause mortality, clinical success, Microbiological eradication, and nephrotoxicity of different regimens used to treat MDR/XDR *Acinetobacter baumannii* pneumonia

Study (year)	Designs	Age (mean ± SD)	N	Infection site	Pathogen (%)	Symbol	Regimen	Severity ^#^ (mean ± SD)	Outcomes (n/N)			
									Mortality	Clinical success	Microbiological eradication	Nephrotoxicity
**Betrosian 2008**	Prospective	67 ± 9	15	VAP	MDR-AB (100)	IV COL mono	IV colistin monotherapy	14 ± 2	5/15	9/15	10/15	5/15
		72 ± 5	13			SUL mono	Sulbactam monotherapy	14 ± 5	3/13	9/13	8/13	2/13
**Bassetti 2021**	RCT	63.1 ± 19.0	101	BSI and pneumonia	CR-GN pathogen (CRAB:64%)	CEF mono	Cefiderocol monotherapy	15.3 ± 6.5	19/45^***^	24/40^***^	12/40^***^	NR
		63.0 ± 16.7	49			IV COL + other	IV colistin + other agent	15.4 ± 6.2	22/4^***^	12/19^***^	5/19^***^	NR
**Chuang 2014**	Retrospective	63.8	84	VAP	MDR-AB (100)	IV COL mono	IV colistin monotherapy	21.6	37/84	NR	NR	NR
		63.5	84			TIG mono	Tigecycline monotherapy	22.0	51/84	NR	NR	NR
**Dalfino 2023**	Prospective	67	40	VAP	MDR-AB (100)	CEF + other	Cefiderocol + other agent	24	14/40	30/40	35/40	NR
		64	50			IV COL mono	IV colistin monotherapy	22	26/50	26/50	40/50	NR
**Deng 2022**	Retrospective	69	24	VAP (or HAP)	MDR-AB (100)	SUL + other	Sulbactam + other agent	NR	3/24	NR	NR	NR
			30			SUL + TIG	Sulbactam + tigecycline		2/30	NR	NR	NR
			23			TIG + other	Tigecycline + other agent		3/23	NR	NR	NR
			2			IV COL mono	IV colistin monotherapy		1/2	NR	NR	NR
**Durante Mangoni 2013**	RCT	61	105	VAP (or HAP)	MDR-AB (100)	IV COL mono	IV colistin monotherapy	39.0^*^	45/105	NR	47/105	29/101
		62	104			IV COL + Other	IV colistin + other agent	40.8^*^	45/104	NR	63/104	24/101
**Falcone 2022**	Retrospective	63	47	BSI (57.4%) and VAP (25.5%) and other (17%)	CRAB (100)	CEF + Other	Cefiderocol + other agent	9^**^	4/12^***^	NR	NR	NR
		68	77			IV COL + Other	IV colistin + other agent	9^**^	12/23^***^	NR	NR	NR
**Hsieh 2014**	Retrospective	82.2 ± 16.1	9	VAP (or HAP)	XDR-AB (100)	IH COL mono	Inhaled colistin monotherapy	16.4 ± 4.6	3/9	NR	7/9	NR
		79.2 ± 9.9	29			IH COL + TIG	Inhaled colistin + tigecycline	17.8 ± 7.3	10/29	NR	23/29	NR
**Kalin 2014**	Retrospective	52	47	VAP	MDR-AB (100)	IV COL mono	IV colistin monotherapy	22	27/47	14/47	34/47	NR
		63	35			IV COL + SUL	IV colistin + sulbactam	27	27/35	14/35	30/35	NR
**Khawcharoenporn 2014**	Retrospective	75	30	VAP (or HAP)	XDR-AB (100)	IH COL + Other	Inhaled colistin + other agent	18	18/30	NR	16/22	2/30
		75	43			IH COL + TIG	Inhaled colistin + tigecycline	20	23/43	NR	25/38	3/43
		75	93			IH COL + SUL	Inhaled colistin + sulbactam	18	60/93	NR	59/70	8/93
**Kim 2016**	Retrospective	67	40	VAP (or HAP)	MDR/XDR-AB (100)	IV COL mono	IV colistin monotherapy	10^**^	12/40	19/40	12/40	8/40
		72	30			TIG mono	Tigecycline monotherapy	9.5^**^	10/30	14/30	7/30	0/30
**Kwon 2014**	Retrospective	59.0 ± 19.2	39	HAP	XDR-AB (100)	IV COL mono	IV colistin monotherapy	NR	17/39	19/39	18/39	17/39
		60.1 ± 12.3	16			TIG mono	Tigecycline monotherapy		9/16	7/16	2/16	2/16
**Pascale 2021**	Retrospective	64	42	BSI (58%) and LRTI (41%)	CRAB (100)	CEF + Other	Cefiderocol + other agent	9^**^	3/15^***^	NR	NR	NR
		65	65			CAR + Other	Carbapenem + other agent	8^**^	5/30^***^	NR	NR	NR
**Rando 2023**	Prospective	64	55	VAP	CRAB (100)	CEF + Other	Cefiderocol + other agent	7^**^	24/55	NR	NR	NR
		68	66			IV COL + Other	IV colistin + other agent	8^**^	44/66	NR	NR	NR
**Russo 2023**	Retrospective	60	54	VAP	MDR-AB (100)	IV COL mono	IV colistin monotherapy	10^**^	53/54	NR	NR	NR
		61	19			CEF + Other	Cefiderocol + other agent	9^**^	6/19	NR	NR	NR
**Ungthammakhun 2019**	Prospective	72.1 ± 16.5	92	VAP (or HAP)	XDR-AB (100)	IV COL + SUL	IV colistin + sulbactam	20.4	47/92	NR	NR	NR
		68.6 ± 18.6	90			IV COL + Other	IV colistin + other agent	21.0	50/90	NR	NR	NR
**Yee 2024**	Retrospective	57.3	1	VAP	MDR/XDR-AB (100)	SUL + Other	Sulbactam + other agent	NR	1/1	NR	NR	NR
			1			SUL + TIG	Sulbactam + tigecycline		0/1	NR	NR	NR
			94			POL + CAR	polymyxin B + carbapenem		64/94	NR	NR	NR
			3			TIG + Other	Tigecycline + other agent		2/3	NR	NR	NR
			16			IV COL + Other	IV colistin + other agent		13/16	NR	NR	NR
			2			SUL mono	Sulbactam monotherapy		0/2	NR	NR	NR
			1			TIG mono	Tigecycline monotherapy		0/1	NR	NR	NR
**Yilmaz 2015**	Retrospective	59.8 ± 21.5	17	VAP	MDR/XDR-AB (100)	IV COL mono	IV colistin monotherapy	43.8 ± 12.1^*^	7/17	13/17	9/17	3/17
		59.6 ± 20.5	33			IV COL + Other	IV colistin + other agent	50.7 ± 12.9^*^	16/33	21/33	21/33	4/33
		70.6 ± 14.7	20			IV COL + SUL	IV colistin + sulbactam	51.0 ± 9.8^*^	14/20	11/20	12/20	2/20
**Zheng 2019**	Retrospective	76.8 ± 14.4	18	VAP (or HAP)	MDR-AB (100)	IV COL mono	IV colistin monotherapy	17.7 ± 5.8	5/18	9/18	9/18	NR
			128			IH COL mono	Inhaled colistin monotherapy		17/128	102/128	95/128	NR
			68			IV COL + Other	IV colistin + other agent		12/68	48/68	41/68	NR
			26			IV COL + SUL	IV colistin + sulbactam		3/26	18/26	20/26	NR
			42			IV COL + TIG	IV colistin + tigecycline		10/42	32/42	31/42	NR

RCT: randomized controlled trial, VAP: ventilator-associated pneumonia, HAP: hospital-acquired pneumonia, MDR: multidrug resistant, XDR: extensively drug resistant, AB: Acinetobacter baumannii, IV: intravenous, IH: inhalation, COL: colistin, SUL: sulbactam 3–9 g/day, TIG: tigecycline (100 mg loading dose followed by 50 mg every 12 h), POL: polymyxin B, CAR: carbapenem, CEF: cefiderocol, mono: monotherapy, LRTI: lower respiratory tract infection, BSI: bloodstream infection, CRAB: carbapenem resistant Acinetobacter baumannii, CR-GN: carbapenem resistant Gram-negative, # APACHE II: Acute Physiology and Chronic Health Evaluation II, * SAPS II: Simplified Acute Physiology Score II (mean ± SD), ** SOFA: Sequential Organ Failure Assessment. *** Nosocomial pneumonia cases from this study were extracted and included in the analysis

**Table 2:** Pairwise comparisons and ranking of all-cause mortality associated with different regimens used to treat MDR/XDR *Acinetobacter baumannii* pneumonia

CEF + other	1.25 [0.20, 7.87]	.	.	.	.	.	.	.	.	.	0.20 [0.09, 0.43]	.	.	.	.	.
1.25 [0.20, 7.87]	**IV COL + CAR**	.	.	.	.	.	.	.	.	.	.	.	.	.	.	.
0.88 [0.10, 7.60]	0.71 [0.04, 11.99]	**SUL + TIG**	2.00 [0.05, 87.86]	0.51 [0.08, 3.08]	0.46 [0.07, 3.05]	.	.	0.47 [0.02, 9.08]	0.07 [0.00, 1.85]	.	0.26 [0.01, 6.06]	.	1.00 [0.02, 56.18]	.	.	.
0.61 [0.10, 3.79]	0.49 [0.04, 6.53]	0.69 [0.06, 7.61]	**SUL mono**	0.30 [0.01, 7.93]	0.17 [0.00, 10.13]	.	.	0.24 [0.02, 3.21]	0.60 [0.09, 4.09]	.	0.13 [0.01, 2.20]	.	0.50 [0.01, 21.97]	.	.	.
0.44 [0.06, 3.15]	0.35 [0.02, 5.20]	0.50 [0.08, 3.01]	0.72 [0.08, 6.70]	**TIG + other**	0.92 [0.16, 5.29]	.	.	0.79 [0.08, 7.62]	0.15 [0.01, 3.55]	.	0.43 [0.03, 5.36]	.	1.67 [0.05, 58.36]	.	.	.
0.43 [0.05, 3.69]	0.35 [0.02, 5.84]	0.49 [0.07, 3.20]	0.71 [0.06, 7.73]	0.99 [0.17, 5.58]	**SUL + other**	.	.	1.42 [0.05, 40.86]	0.14 [0.01, 3.38]	.	0.78 [0.0, 26.65]	.	3.00 [0.04,228.92]	.	.	.
0.30 [0.08, 1.13]	0.24 [0.02, 2.31]	0.34 [0.04, 3.20]	0.49 [0.07, 3.31]	0.68 [0.08, 5.46]	0.69 [0.07, 6.49]	**IH COL mono**	0.95 [0.15, 5.96]	.	0.40 [0.09, 1.75]	.	0.71 [0.21, 2.44]	.	.	1.17 [0.24, 5.84]	0.49 [0.14, 1.76]	.
0.28 [0.03, 2.73]	0.23 [0.01, 4.20]	0.32 [0.02, 5.85]	0.46 [0.03, 6.57]	0.65 [0.04, 10.38]	0.65 [0.04, 11.87]	0.95 [0.15, 5.96]	**IH COL + TIG**	.	.	0.77 [0.20, 2.88]	.	0.63 [0.19, 2.07]	.	.	.	.
0.29 [0.06, 1.38]	0.23 [0.02, 2.59]	0.33 [0.05, 2.36]	0.48 [0.07, 3.05]	0.66 [0.11, 3.87]	0.67 [0.09, 4.84]	0.98 [0.18, 5.37]	1.03 [0.08, 12.59]	**POL + CAR**	.	.	0.55 [0.12, 2.61]	.	2.11 [0.11, 40.60]	.	.	.
0.24 [0.09, 0.68]	0.19 [0.02, 1.59]	0.27 [0.04, 2.05]	0.40 [0.08, 1.92]	0.55 [0.09, 3.43]	0.56 [0.08, 4.15]	0.81 [0.26, 2.56]	0.86 [0.10, 7.46]	0.83 [0.20, 3.40]	**IV COL mono**	.	1.08 [0.49, 2.36]	.	0.62 [0.29, 1.31]	0.60 [0.24, 1.48]	1.23 [0.26, 5.85]	.
0.22 [0.02, 3.00]	0.17 [0.01, 4.29]	0.25 [0.01, 5.98]	0.35 [0.02, 6.89]	0.50 [0.02, 10.74]	0.50 [0.02, 12.15]	0.73 [0.08, 7.01]	0.77 [0.20, 2.88]	0.75 [0.04, 12.70]	0.90 [0.07, 11.33]	**IH COL + other**	.	0.82 [0.23, 2.90]	.	.	.	.
0.20 [0.09, 0.43]	0.16 [0.02, 1.17]	0.23 [0.03, 1.69]	0.33 [0.06, 1.71]	0.46 [0.07, 2.81]	0.46 [0.06, 3.42]	0.67 [0.23, 1.99]	0.70 [0.08, 5.96]	0.69 [0.18, 2.66]	0.82 [0.42, 1.63]	0.92 [0.07, 11.34]	**IV COL + other**	.	3.86 [0.17, 90.09]	0.96 [0.44, 2.09]	0.69 [0.18, 0.58]	0.30 [0.06, 1.43]
0.18 [0.01, 2.31]	0.14 [0.01, 3.35]	0.20 [0.01, 4.67]	0.29 [0.02, 5.35]	0.41 [0.02, 8.36]	0.41 [0.02, 9.48]	0.60 [0.07, 5.34]	0.63 [0.19, 2.07]	0.62 [0.04, 9.84]	0.74 [0.06, 8.71]	0.82 [0.23, 2.90]	0.90 [0.08, 10.32]	**IH COL + SUL**	.	.	.	.
0.16 [0.05, 0.57]	0.13 [0.01, 1.21]	0.19 [0.02, 1.53]	0.27 [0.05, 1.50]	0.38 [0.05, 2.58]	0.38 [0.05, 3.10]	0.55 [0.14, 2.13]	0.58 [0.06, 5.67]	0.57 [0.12, 2.60]	0.68 [0.33, 1.41]	0.76 [0.05, 0.57]	0.82 [0.31, 2.19]	0.92 [0.07, 11.98]	**TIG mono**	.	.	.
0.17 [0.06, 0.47]	0.13 [0.02, 1.10]	0.19 [0.02, 1.52]	0.27 [0.05, 1.52]	0.38 [0.06, 2.55]	0.38 [0.05, 3.07]	0.56 [0.17, 1.79]	0.59 [0.07, 5.17]	0.57 [0.13, 2.52]	0.68 [0.31, 1.49]	0.76 [0.06, 9.77]	0.83 [0.41, 1.69]	0.93 [0.08, 11.04]	1.01 [0.35, 2.89]	**IV COL + SUL**	0.42 [0.08, 2.23]	.
0.15 [0.04, 0.60]	0.12 [0.01, 1.19]	0.17 [0.02, 1.66]	0.24 [0.03, 1.73]	0.33 [0.04, 2.83]	0.34 [0.03, 3.36]	0.49 [0.14, 1.76]	0.52 [0.06, 4.82]	0.50 [0.09, 2.96]	0.60 [0.17, 2.10]	0.67 [0.05, 9.05]	0.73 [0.22, 2.42]	0.82 [0.06, 10.24]	0.89 [0.21, 3.74]	0.88 [0.25, 3.13]	**IV COL + TIG**	.
0.06 [0.01, 0.34]	0.05 [0.00, 0.60]	0.07 [0.01, 0.87]	0.10 [0.01, 0.95]	0.14 [0.01, 1.51]	0.14 [0.01, 1.76]	0.20 [0.03, 1.35]	0.21 [0.02, 2.99]	0.21 [0.03, 1.63]	0.25 [0.05, 1.35]	0.28 [0.01, 5.34]	0.30 [0.06, 1.43]	0.34 [0.02, 6.09]	0.37 [0.06, 2.30]	0.37 [0.07, 2.00]	0.42 [0.06, 2.93]	**CEF mono**

Antimicrobial regimens were ranked based on reducing all-cause mortality. The CEF + other ranked highest, followed by IV COL + CAR and SUL + TIG. For a comprehensive ranking of regimens, refer to Table 2.

**Fig. 4:**
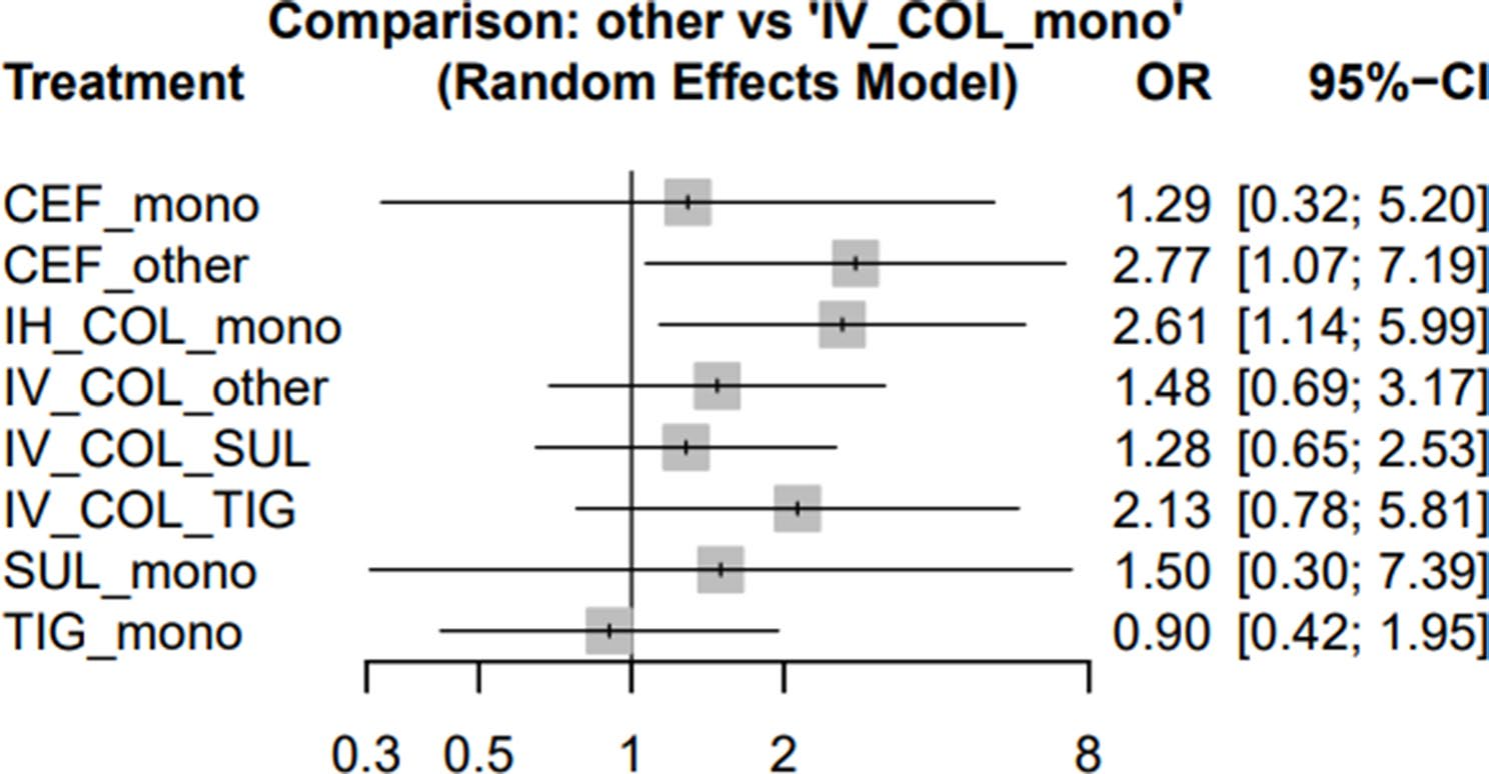
Forest plots illustrating the odds ratios (ORs) for clinical success associated with different regimens used to treat MDR/XDR *Acinetobacter baumannii* pneumonia

### Secondary outcomes: clinical success, microbiological eradication, and nephrotoxicity

Regarding clinical success, CEF + other (OR 2.77; 95% CI 1.07–7.19) and IH COL mono (OR 2.61; 95% CI 1.14–5.99) were associated with significantly higher odds of symptom resolution compared with IV colistin monotherapy. In contrast, CEF mono (OR 1.29; 95% CI 0.32–5.20), IV COL + other (OR 1.48; 95% CI 0.69–3.17), IV COL + SUL (OR 1.28; 95% CI 0.65–2.53), IV COL + TIG (OR 2.13; 95% CI 0.78–5.81), SUL mono (OR 1.50; 95% CI 0.30–7.39), and TIG mono (OR 0.90; 95% CI 0.42–1.95) did not demonstrate statistically significant differences (Fig. 4). Full ranking results are provided in Supplementary Table S3.

Microbiological eradication findings are summarized in Figure S1. Compared with IV COL mono, several regimens showed significantly higher eradication rates, including IH COL mono (OR: 2.95, 95% CI: 1.54–5.65), IV COL + SUL (OR: 2.34, 95% CI: 1.22–4.51), IV COL + TIG (OR: 2.89, 95% CI: 1.22–6.82), and IV COL + other (OR: 1.74, 95% CI: 1.12–2.70). Other regimens such as CEF mono or combination therapy, IH COL based combinations, SUL mono, and TIG mono did not differ significantly from IV COL mono.

Full effect size rankings for all evaluated regimens are provided in Supplementary Table S4. Detailed pairwise comparisons derived from direct evidence are shown in Supplementary Figures S2–S4.

**Fig. 5:**
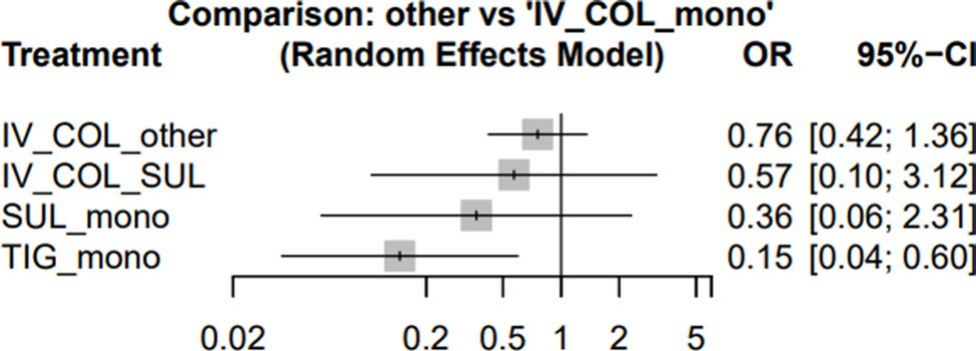
Forest plots illustrating the odds ratios (ORs) for nephrotoxicity associated with different regimens used to treat MDR/XDR *Acinetobacter baumannii* pneumonia

For nephrotoxicity, TIG mono demonstrated a significantly lower risk compared with IV colistin monotherapy (OR 0.15; 95% CI 0.04–0.60). In contrast, SUL mono (OR 0.36; 95% CI 0.06–2.31), IV COL + SUL (OR 0.57; 95% CI 0.10–3.12), and IV COL + other (OR 0.76; 95% CI 0.42–1.36) did not show statistically significant differences (Fig. 5). Full nephrotoxicity rankings are provided in Table S5.

### Inconsistency test

A network was constructed with nodes representing different regimens, enabling both direct and indirect comparisons. Inconsistency testing for all-cause mortality yielded *p*-values > 0.05, indicating no significant inconsistencies across the network. The heterogeneity assessment demonstrated an I² of 68.8% and a τ² of 0.578, reflecting moderate between-study variability. Inconsistency analyses for all-cause mortality, clinical success, microbiological eradication, and nephrotoxicity are provided in Supplementary Tables S6-S9.

### Sensitivity analyses

The one study removed sensitivity analysis confirmed the stability of the treatment rankings. CEF + other remained significantly associated with reduced all-cause mortality across all models (Supplementary Figure S5). Additional sensitivity analyses for clinical success, microbiological eradication, and nephrotoxicity are provided in Figures S6–S8.

### Publication bias

The funnel plot is shown in (Supplementary Figure S9). Egger’s test yielded a *p*-value of 0.457, suggesting no significant publication bias.

## Discussion

Our network meta-analysis comprehensively evaluated the comparative efficacy and safety of various antimicrobial regimens for the treatment of pneumonia caused by MDR/XDR-AB. The clinical severity of illness did not differ significantly between the VAP and HAP groups in our cohort. Among all evaluated therapies, cefiderocol containing regimens (OR 0.24; 95% CI 0.09–0.68) were associated with the most notable reduction in all-cause mortality, indicating a possible benefit compared with intravenous colistin and other regimens. Additionally, cefiderocol containing regimens (OR 2.77; 95% CI 1.07–7.19) and inhaled colistin (OR 2.61; 95% CI 1.14–5.99) were both associated with significantly higher clinical success rates, as reflected by resolution of pneumonia related symptoms. In terms of microbiological eradication, inhaled colistin and IV colistin in combination with sulbactam or tigecycline showed notable efficacy, suggesting potential roles for these regimens in achieving pathogen clearance.

However, nephrotoxicity remains a concern, particularly with IV colistin-based regimens. Tigecycline monotherapy (OR 0.15; 95% CI 0.04–0.60) was the only regimen associated with a significantly lower risk of nephrotoxicity. Our study provides updated comparative data on traditional and novel treatment options for MDR/XDR-AB pneumonia, and supports the potential role of cefiderocol combination regimes as a preferred therapeutic agent, while also reaffirming the importance of evaluating safety and efficacy tradeoffs when selecting antimicrobial strategies.

XDR- and MDR-AB have emerged as critical pathogens in HAP and VAP. According to the report by the previous study, AB, particularly carbapenem resistant strains, ranks among the top causes of nosocomial pneumonia in ICU worldwide [41]. In many hospitals, especially in Asia and southern Europe, MDR- and XDR-AB pneumonia account for a significant proportion of HAP and VAP, leading to high morbidity, prolonged hospitalization, and increased mortality [9, 42]. The high adaptability of AB in hospital environments and its capability to acquire resistance mechanisms contribute to its endemic status in ICUs.

Intravenous colistin has traditionally been used therapy against MDR/XDR-AB. However, its clinical application is hindered by its poor lung parenchymal penetration and significant nephrotoxicity. A pharmacokinetic study by showed that IV colistin achieves suboptimal concentrations in epithelial lining fluid (ELF), which may be inadequate for eradicating pathogens in the alveolar space [43]. Moreover, the previous studies highlighted that nephrotoxicity rates range between 30 and 60%, often limiting its use, particularly in patients with pre-existing renal impairment [44–46]. These limitations have led clinicians to explore alternative administration routes and combination strategies in pneumonia treatment. Inhaled colistin has emerged as a promising adjunct or alternative to IV administration for MDR/XDR-AB pneumonia. Several observational studies and small randomized controlled trials have shown that Inhaled colistin can achieve higher ELF concentrations, thereby overcoming the limitation of poor lung tissue penetration seen with IV use. The study reported improved clinical and microbiological outcomes in patients receiving inhalation colistin in combination with systemic antibiotics [47]. Nonetheless, challenges remain. The optimal dosing, nebulizer system, and standardization of IH formulations vary widely across institutions. Additionally, the potential for bronchospasm and aerosol dispersion raises concerns for both patient safety and healthcare worker exposure.

Tigecycline and sulbactam remain important therapeutic options in the management of MDR/XDR AB pneumonia, particularly in regions where alternative agents are limited. Tigecycline, a glycylcycline with broad intracellular penetration, exhibits in vitro activity against MDR AB and has been widely used as part of combination therapy to enhance antibacterial synergy. However, concerns regarding suboptimal serum and ELF concentrations have been consistently reported, especially in critically ill patients, raising questions about its adequacy as monotherapy for severe pneumonia [48]. High dose tigecycline regimens have been explored to overcome these pharmacokinetic limitations, yet randomized evidence remains insufficient and observational studies have yielded inconsistent clinical outcomes [49]. Sulbactam, a β-lactamase inhibitor with intrinsic activity against AB, has demonstrated meaningful efficacy when administered at high doses, often as part of combination therapy with colistin or carbapenems. The need for high pharmacodynamic target attainment, particularly in isolates with elevated MICs, underscores the importance of optimized dosing strategies [50]. Despite supportive real-world data, sulbactam monotherapy is generally inadequate in severe infections, and its effectiveness appears highly dependent on both pathogen susceptibility and adequate source control [51].

The debate on monotherapy versus combination therapy in treating MDR/XDR-AB pneumonia remains unresolved. One of the meta-analyses found no clear mortality benefit for combination therapy over monotherapy; however, subgroup analyses suggest that certain combinations may enhance microbiological eradication and clinical outcomes [52]. Combinations involving high dose sulbactam, tigecycline, polymyxin B, and inhalation colistin have shown synergistic activity in vitro and in limited clinical studies. For example, the previous studies demonstrated improved outcomes when tigecycline was combined with inhalation colistin or polymyxin B. Moreover, sulbactam’s intrinsic activity against AB, especially at high doses, makes it a valuable partner [53, 54]. Nevertheless, inconsistencies in dosing, regional resistance patterns, and lack of large scale RCTs hinder definitive conclusions. To date, no well-established guidelines exist regarding the optimal combination regimen and appropriate dosing strategies.

Cefiderocol, a novel siderophore cephalosporin, has garnered attention for its potent activity against carbapenem resistant Gram-negative bacteria (CR-GNB), including AB. Cefiderocol’s may reflect its potent activity against carbapenemase producing AB, enhanced siderophore-mediated uptake, and reliable PK/PD target attainment in critically ill patients. The CREDIBLE-CR trial showed that cefiderocol had non-inferior efficacy to best available therapy in treating CR-GNB infections. However, subgroup analysis raised concerns regarding mortality in the AB subgroup, which warrants caution [37]. Despite this, real world data suggest that cefiderocol may still be a viable option, especially when other agents are contraindicated or fail. Its favorable safety profile and IV only administration make it attractive, though resistance emergence during therapy has been reported [55]. Despite its promising in vitro activity against carbapenem resistant Gram-negative pathogens, including AB, the emergence of resistance during cefiderocol therapy has raised growing concerns. Several case reports and observational studies have documented rapid development of resistance following monotherapy, particularly in critically ill patients with prolonged treatment courses. Mechanisms implicated include mutations in siderophore receptor genes (e.g., *piuA*, *pirA*) and overexpression of β-lactamases such as PER and NDM type enzymes, which may compromise drug uptake and hydrolyze cefiderocol, respectively [56]. In a subset analysis of the CREDIBLE-CR trial, patients with AB infections showed both higher mortality and higher rates of resistance emergence compared to other pathogens. The CREDIBLE-CR trial supplementary data shows that AB in the cefiderocol group exhibited the highest frequency of MIC elevation, with 5 of 12 isolates developing a ≥ 4-fold increase substantially more than other pathogens, including *Pseudomonas aeruginosa* (3 isolates), *Klebsiella pneumoniae* (3 isolates), and *Stenotrophomonas maltophilia* (1 isolate). Notably, the time to resistance emergence was also shortest in AB, with MIC increases occurring at a mean of 9 days after treatment initiation, compared with 13 days for *Klebsiella pneumoniae*, 13.6 days for *Pseudomonas aeruginosa*, and 14 days for *Stenotrophomonas maltophilia*. These findings suggest that AB not only develops cefiderocol resistance more frequently but also more rapidly than other carbapenem-resistant Gram-negative pathogens, highlighting it may have unique propensity for adaptive resistance during therapy.

Interestingly, despite the concerning mortality signal observed in the AB subgroup of the CREDIBLE-CR trial where 83% of cefiderocol-treated cases received monotherapy emerging evidence from real world studies suggests a different trend when cefiderocol is used as part of combination therapy. Recent meta-analyses and cohort evaluations have reported that cefiderocol-containing combination regimens may reduce mortality in MDR/XDR-AB infections, particularly in critically ill patients [57, 58]. Combination therapy may enhance bacterial killing and reduce cefiderocol resistance by suppressing resistant subpopulations and providing complementary mechanisms of action. Evidence from in vitro synergy studies demonstrates that pairing cefiderocol with other active agents can prevent rapid resistance emergence and improve overall bacterial clearance [59]. This highlights the need for cautious use of cefiderocol, preferably as part of a combination regimen in high-risk patients, as well as the importance of routine susceptibility monitoring during treatment. Further studies are warranted to clarify predictive markers of resistance and to guide optimal therapeutic strategies that preserve cefiderocol’s utility against XDR organisms.

Recent advances in drug development have expanded the therapeutic arsenal against MDR/XDR-AB. Novel agents like sulbactam-durlobactam, currently undergoing phase III trials, show promise due to their enhanced β-lactamase inhibition and AB specific activity [60]. Additionally, new inhaled options are under investigation, particularly aminoglycosides such as inhalation amikacin liposomal formulations, which offer high pulmonary concentrations with limited systemic exposure. These formulations may provide an alternative to colistin-based inhaled regimens, with potentially fewer renal and pulmonary side effects [61].

Our study has several limitations that warrant consideration. First, the minimum inhibitory concentrations (MICs) of MDR/XDR-AB strains were not uniformly reported or stratified across the included studies, which may influence the interpretation of antimicrobial efficacy. Second, dosing regimens for each antibiotic varied among studies, and not all followed standardized or high dose strategies, potentially affecting treatment outcomes and comparability. Third, the analysis included both randomized controlled trials and observational studies, introducing heterogeneity and potential bias in study quality. Fourth, while inhaled colistin was incorporated into the network, other inhaled antibiotics such as aminoglycosides were not included due to limited data availability, potentially underrepresenting inhalation therapy options. Fifth, some newly developed antibiotics, including sulbactam-durlobactam and other investigational agents, were excluded because clinical trial data remain unpublished or insufficient for analysis. Sixth, several treatment comparisons demonstrated wide confidence intervals, reflecting substantial imprecision in the effect estimates. This imprecision is likely attributable to the scarcity of eligible studies informing specific network nodes and the small sample sizes of the included cohorts. Finally, the predominance of small, underpowered studies in the network may introduce small study bias, including possible overestimation of treatment effects and reduced robustness of the pooled estimates. These limitations suggest that, while the findings offer valuable insights, they should be interpreted with caution, and further high quality, standardized research is needed.

The treatment of pneumonia caused by MDR/XDR-AB remains a significant challenge in clinical practice. While inhaled colistin, combination regimens, and newly developed agents offer promising options, there is still a critical need for standardized, high-quality evidence to guide optimal therapeutic strategies. Our study represents the first network meta-analysis (NMA) to focus on MDR/XDR-AB HAP or VAP, excluding other infection sites such as bacteremia or urinary tract infections. It is also the study to limit the analysis solely to AB, rather than including other gram-negative pathogens, thereby providing more pathogen specific insights. Notably, this study is the first to incorporate the novel agent cefiderocol into a comparative effectiveness framework alongside both traditional and inhaled therapies. By employing a network meta-analysis, our study found that cefiderocol containing regimens were associated with the greatest reduction in mortality among patients with MDR/XDR-AB VAP and HAP. This approach allowed for the comparative ranking of treatments, offering valuable insights to support evidence-based clinical decision making.

## Conclusions

Our study provides comprehensive evidence on current and emerging treatments for MDR/XDR-AB pneumonia. Among the available regimens, cefiderocol-containing regimens appear to be the most effective in reducing mortality and improving clinical outcomes. The risk of nephrotoxicity with colistin-based therapies should be carefully considered when selecting these treatment options. However, larger, adequately powered randomized controlled trials are needed to validate these mortality benefits, better define the optimal clinical context for cefiderocol use, and determine whether combination strategies provide additional advantages over monotherapy in severe MDR/XDR-AB pneumonia.

## Supplementary Information

Below is the link to the electronic supplementary material.

Supplementary Material 1

## Data Availability

Data relevant to the findings of this study are available in the article and its supplementary files.

## Abbreviations

AB: Acinetobacter baumannii
CAR: Carbapenem
CEF: Cefiderocol
COL (IH/IV): Colistin (inhaled/intravenous)
CRAB: Carbapenem-resistant Acinetobacter baumannii
CR-GNB: Carbapenem-resistant Gram-negative bacteria
ELF: Epithelial lining fluid
ESCMID: European Society of Clinical Microbiology and Infectious Diseases
GNB: Gram-negative bacilli
GPC: Gram-positive cocci
HAP: Hospital-acquired pneumonia
HD: High dose
ICU: Intensive care unit
IDSA: Infectious Diseases Society of America
IH: Inhaled
IV: Intravenous
MIC: Minimum inhibitory concentration
MDR: Multidrug-resistant
NMA: Network meta-analysis
NOS: Newcastle–Ottawa Scale
OR: Odds ratio
POL: Polymyxin
PRISMA: Preferred Reporting Items for Systematic Reviews and Meta-Analyses
RCT: Randomized controlled trial
SUL: Sulbactam
TIG: Tigecycline
VAP: Ventilator-associated pneumonia
XDR: Extensively drug-resistant
