# Supplementary material for: Comparative effectiveness of antimicrobial regimens for pneumonia caused by drug-resistant Acinetobacter baumannii: a network meta-analysis including cefiderocol and inhaled therapies
Source: BMC Infect Dis. 2026 Jan 20;26:350. doi: 10.1186/s12879-026-12640-z (PMC12905961; doi:10.1186/s12879-026-12640-z)
Supplement: Supplementary file 1 — Supplementary Material 1 [file 12879_2026_12640_MOESM1_ESM.docx]

**Supplementary data**

Figure S1: Forest plots illustrating the odds ratios (ORs) for microbiological eradication associated with different regimens used to treat MDR/XDR *Acinetobacter baumannii* pneumonia.


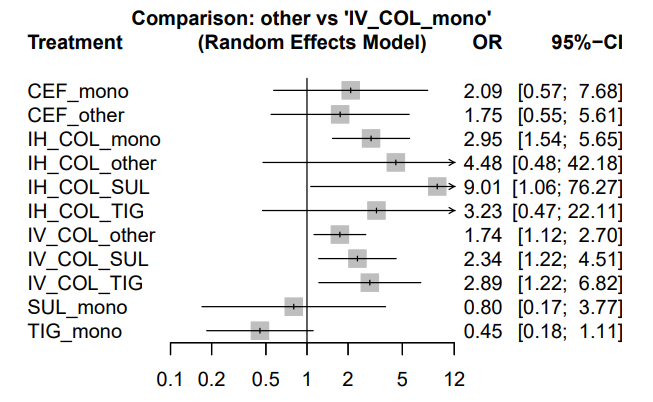


Figure S2: The forest plot of pairwise comparisons among different regimens for treating MDR/XDR *Acinetobacter baumannii* pneumonia, based on the included trials, illustrates the odds ratios (ORs) for all- cause mortality.


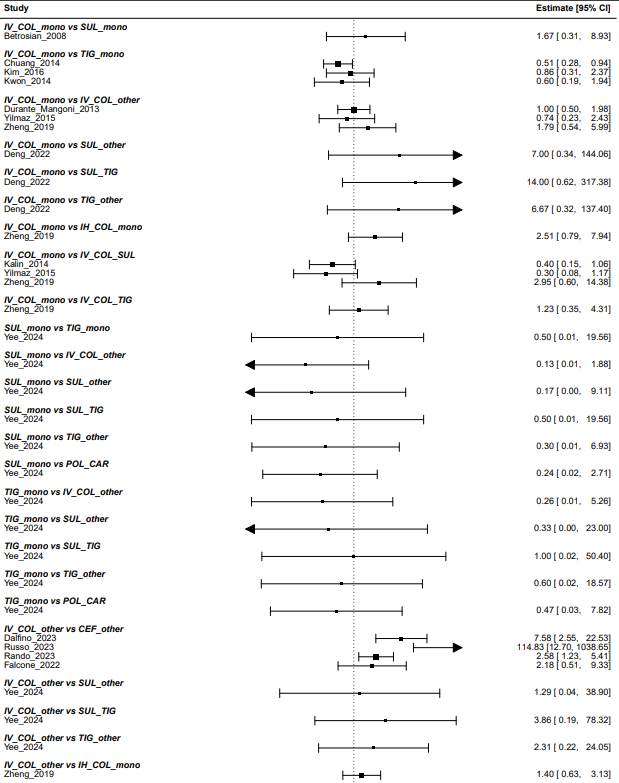


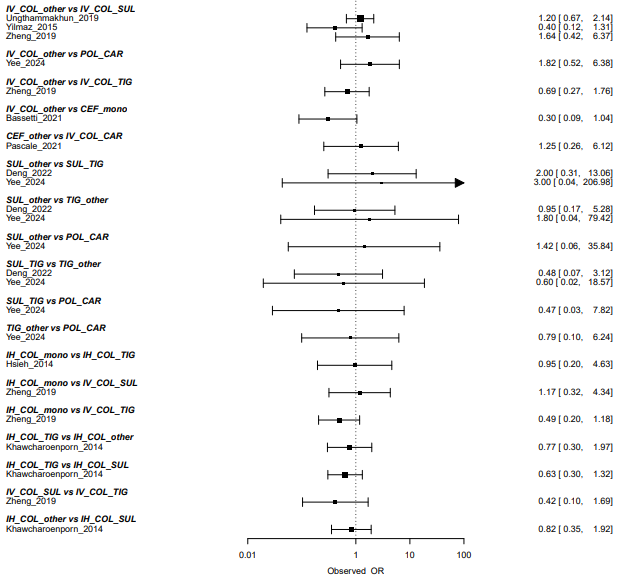


Figure S3: The forest plot of pairwise comparisons among different regimens for treating MDR/XDR *Acinetobacter baumannii* pneumonia, based on the included trials, illustrates the odds ratios (ORs) for clinical success.


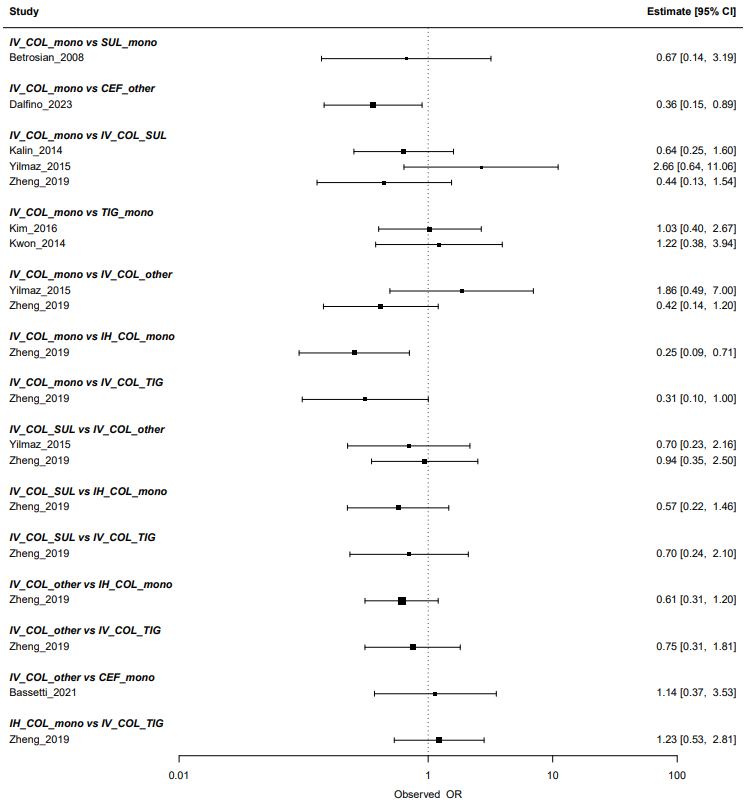


Figure S4: The forest plot of pairwise comparisons among different regimens for treating MDR/XDR *Acinetobacter baumannii* pneumonia, based on the included trials, illustrates the odds ratios (ORs) for microbiological eradication.


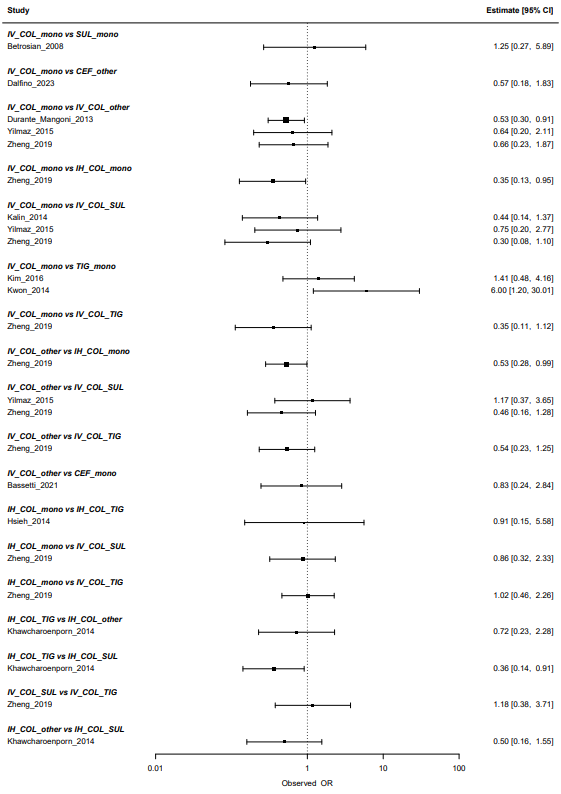


Figure S5: Forest plots from the sensitivity analysis of all-cause mortality, using the one-study removal method, are presented. Each of the 19 included studies was sequentially excluded from the analysis, and the corresponding plots are labeled from A to Q.


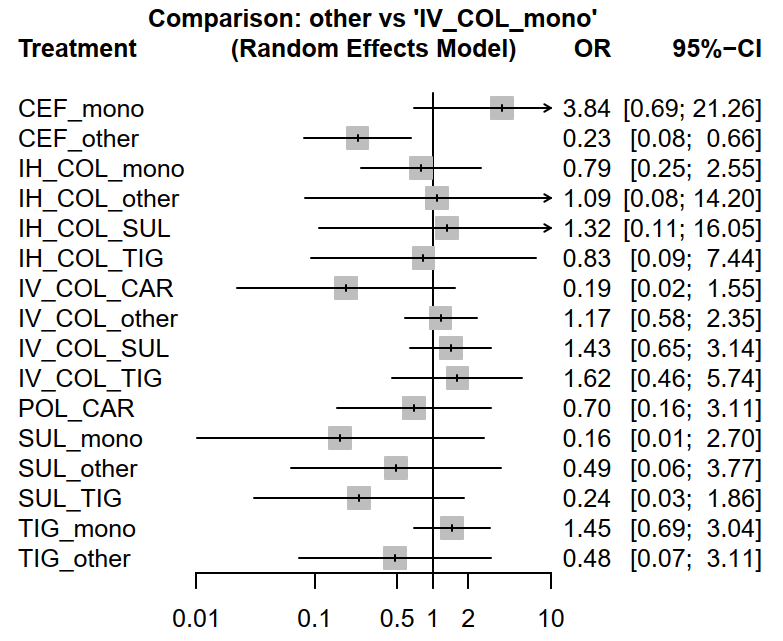


(A)


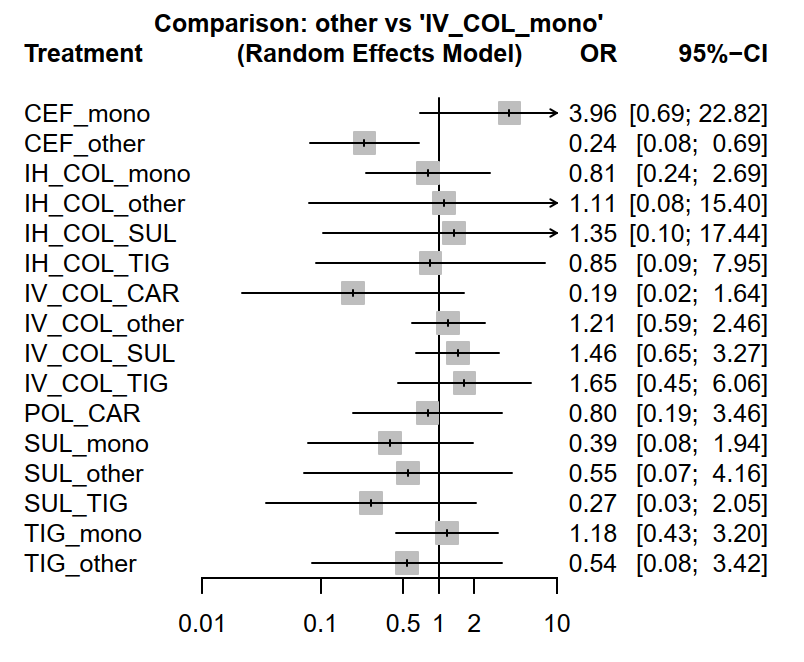


(B)


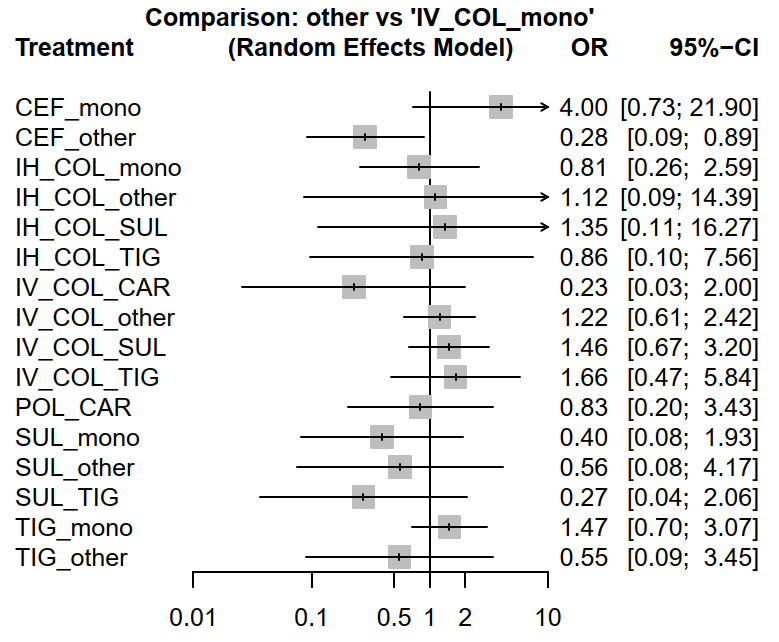


(C)


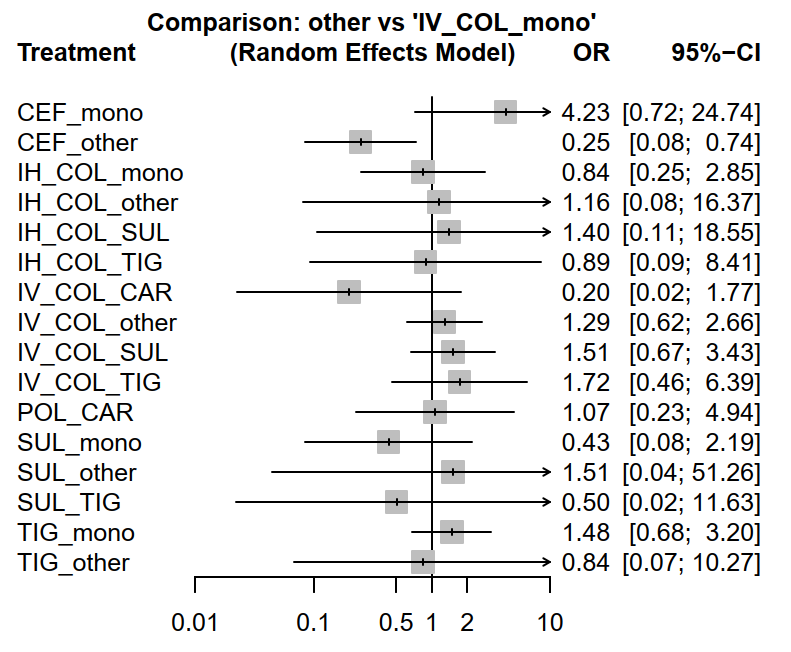


(D)


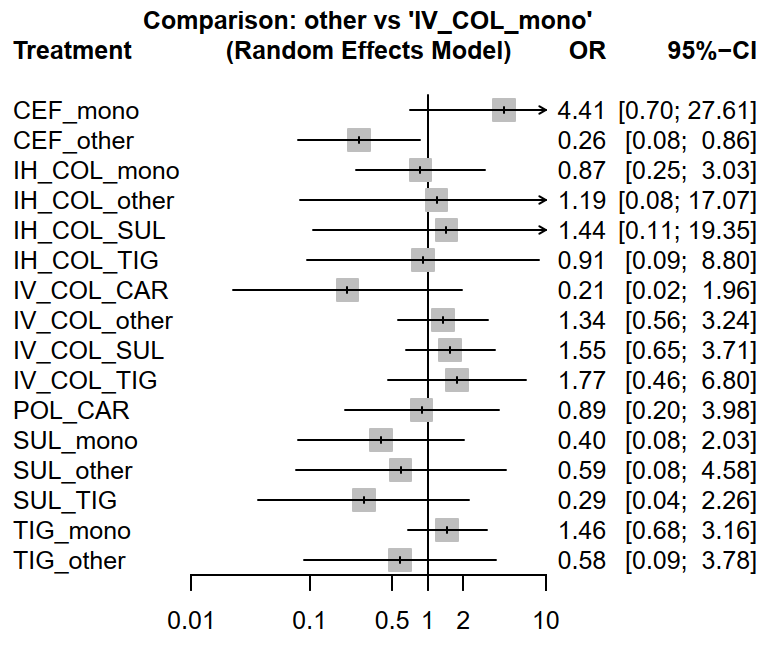


(E)


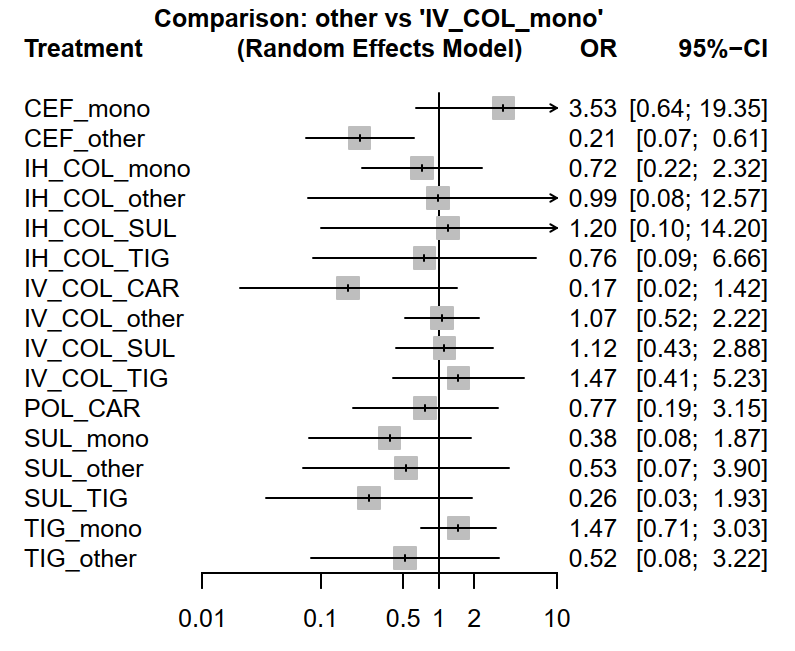


(F)


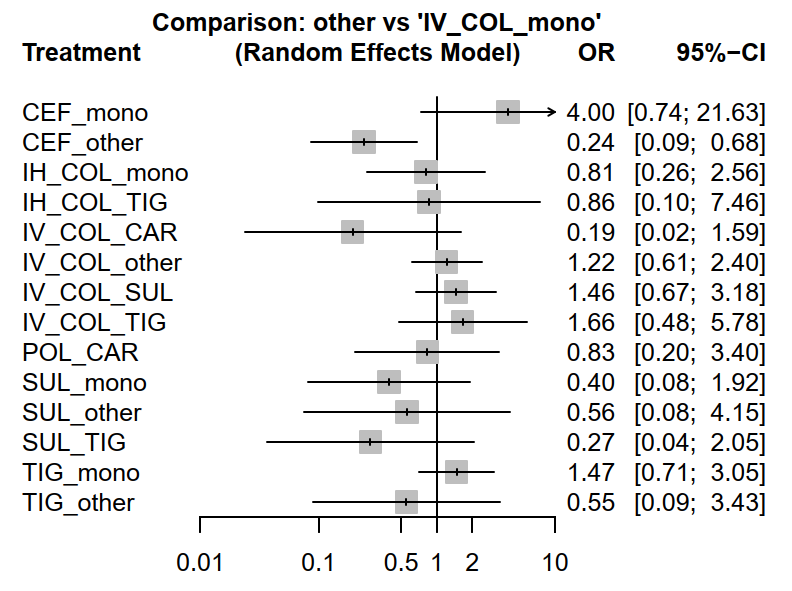


(G)


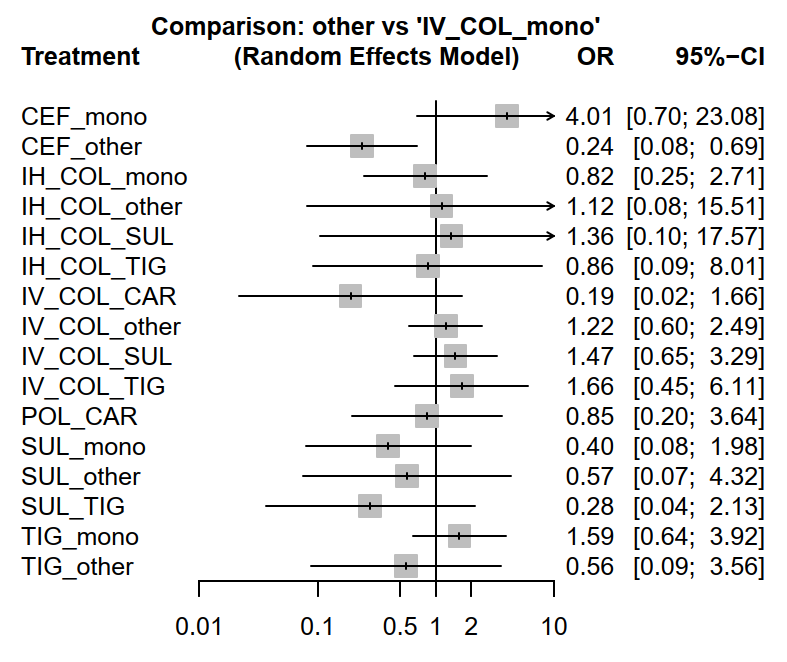


(H)


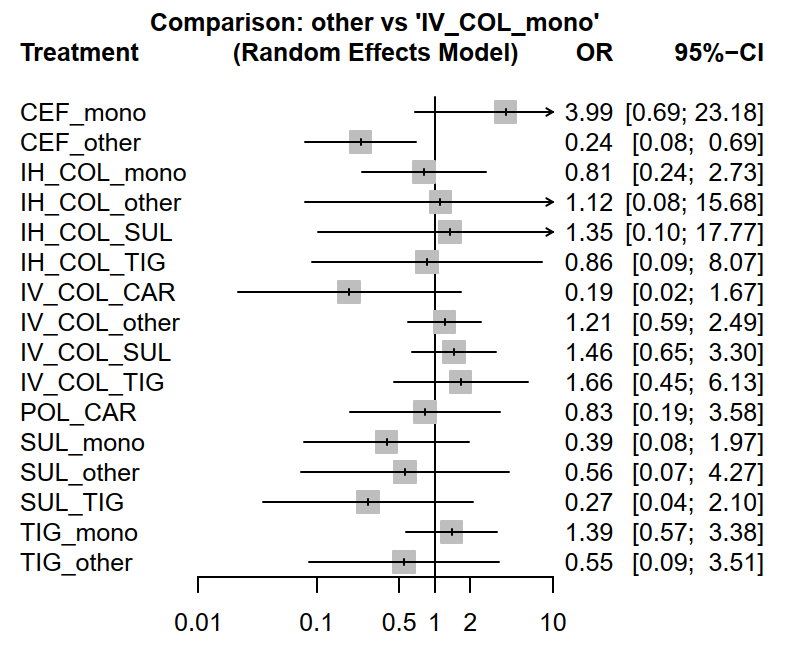


(I)


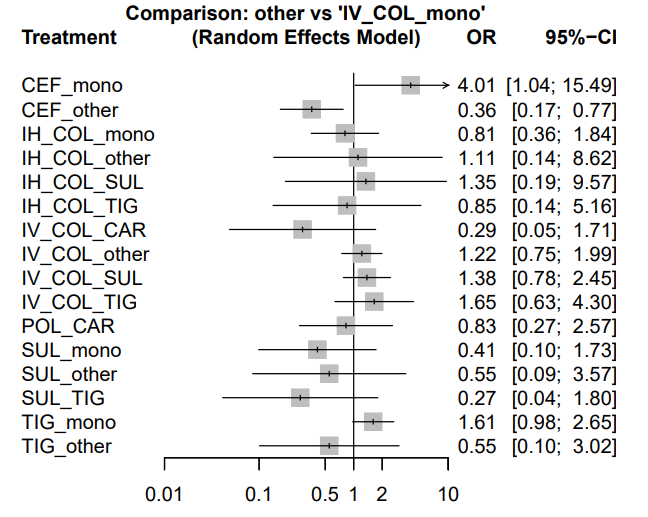


(J)


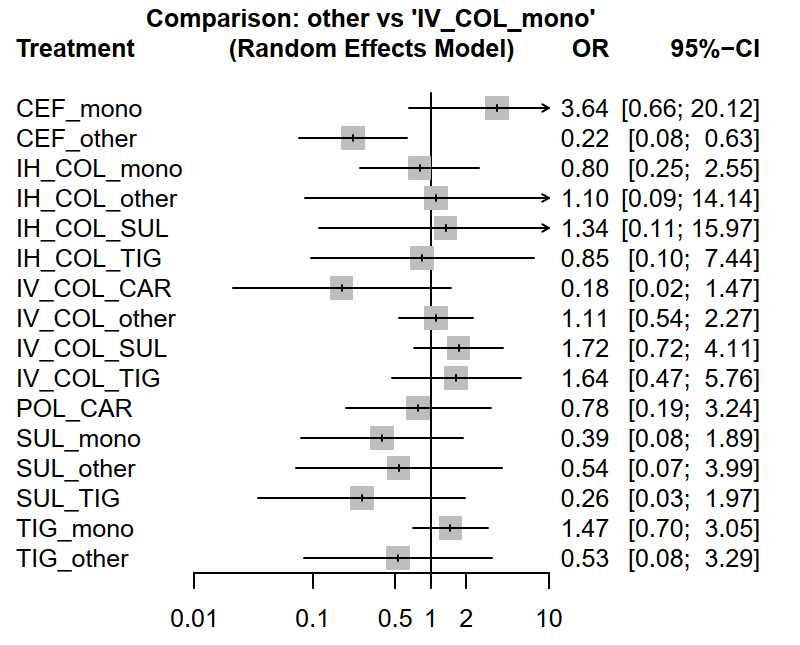


(K)


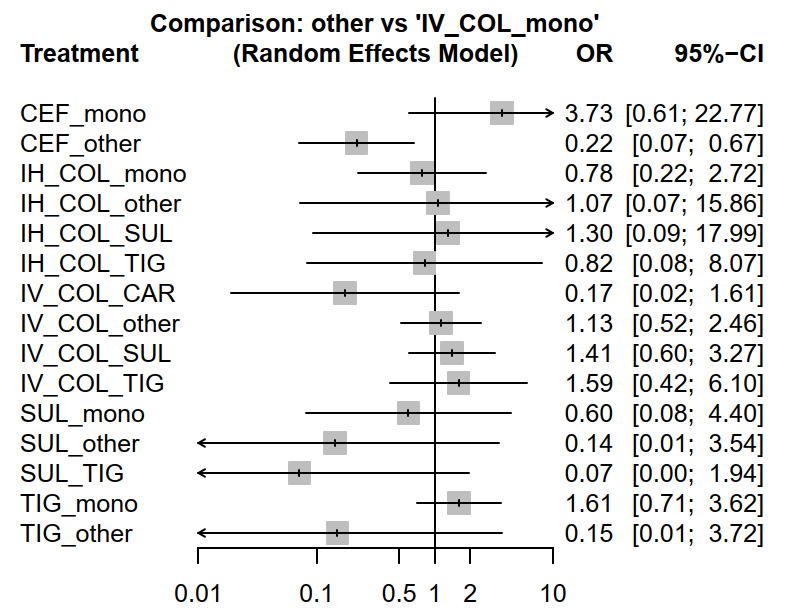


(L)


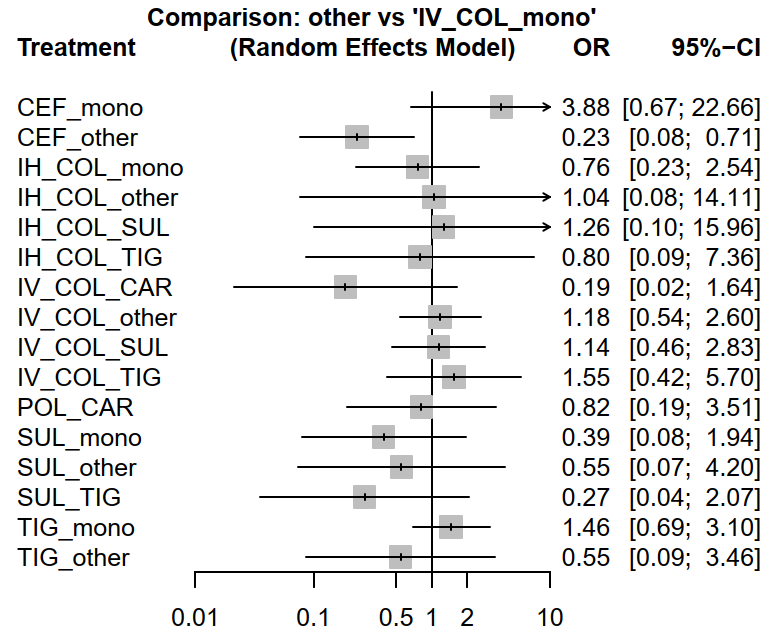


(M)


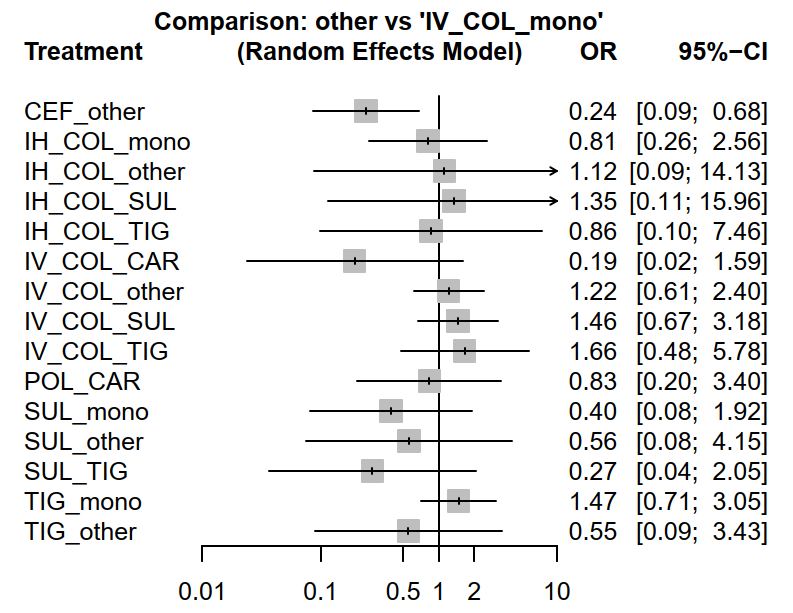


(N)


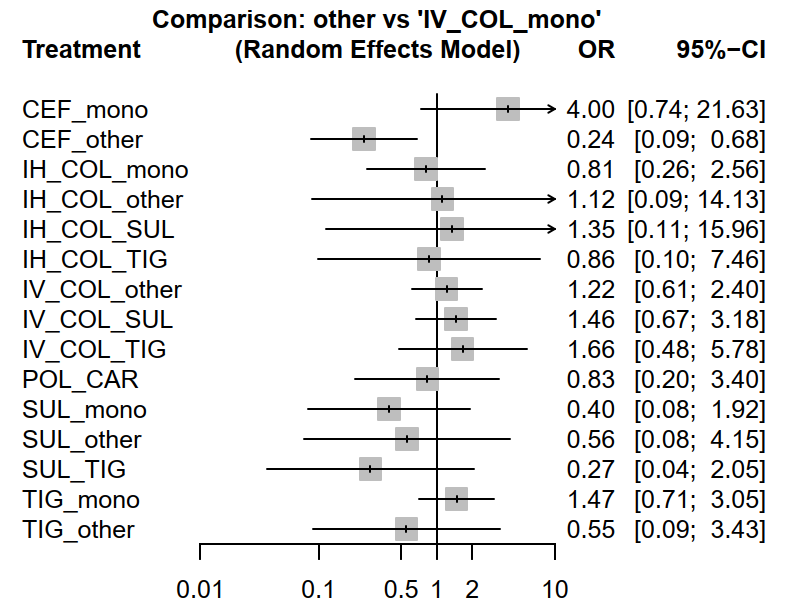


(O)


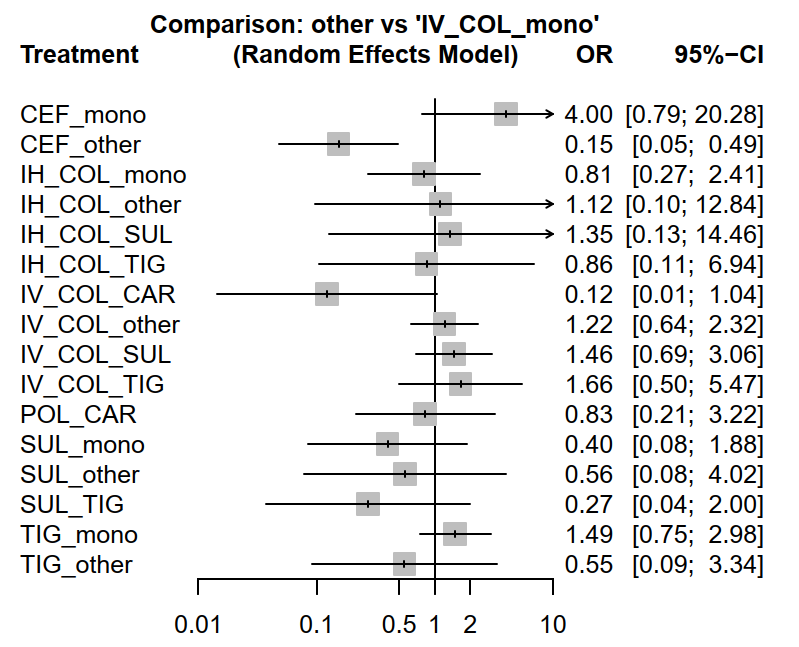


(P)


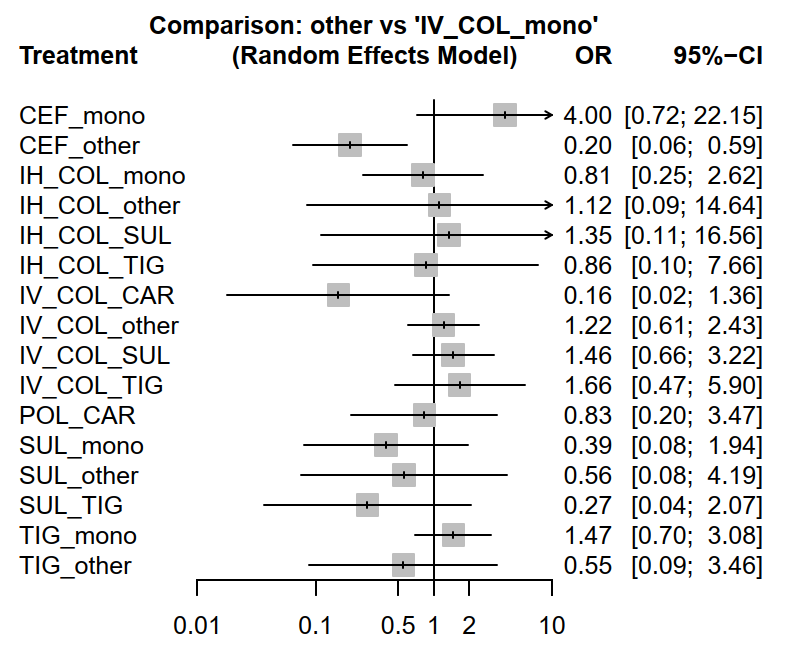


(Q)

Figure S6: Forest plots from the sensitivity analysis of clinical success, using the one-study removal method, are presented. Each of the 8 included studies was sequentially excluded from the analysis, and the corresponding plots are labeled from A to H.


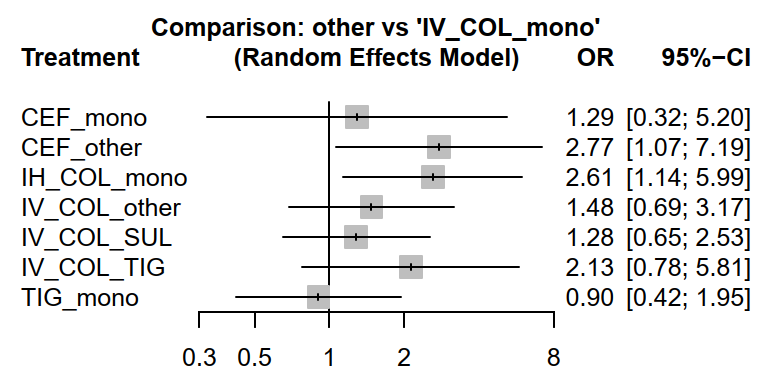


(A)


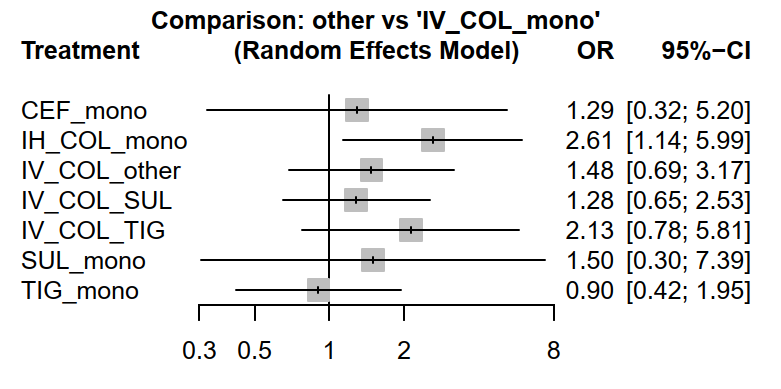


(B)


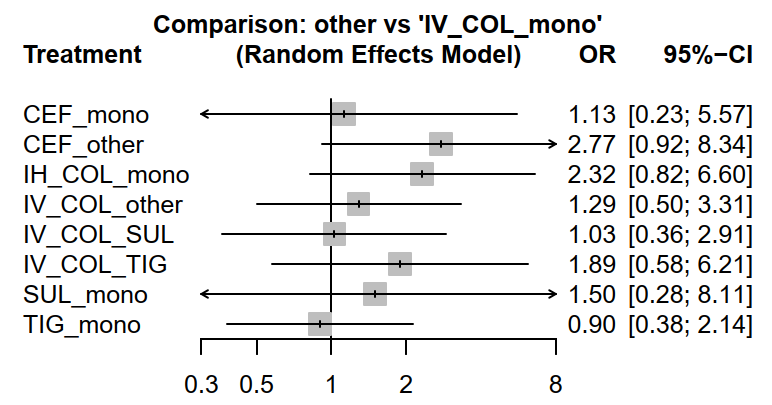


(C)


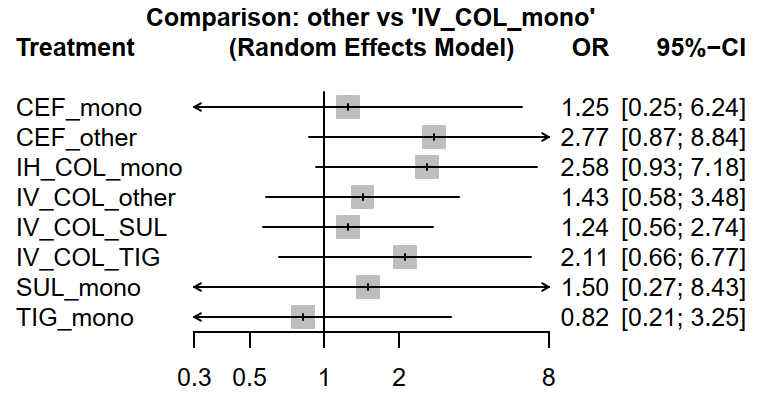


(D)


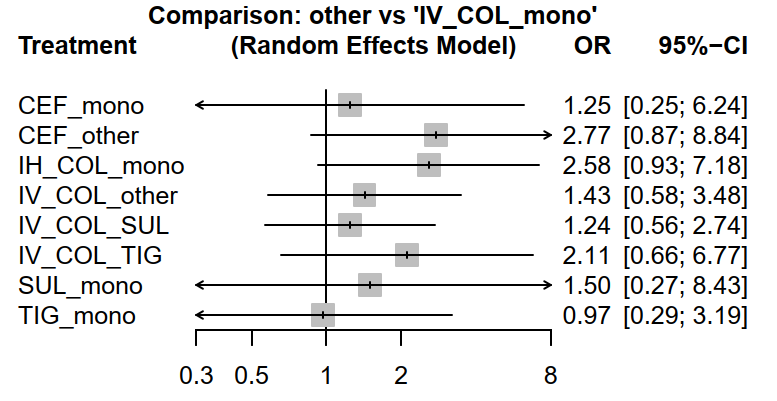


(E)


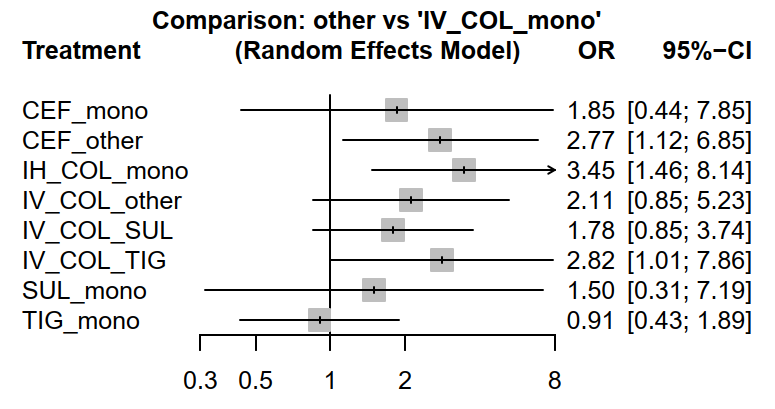


(F)


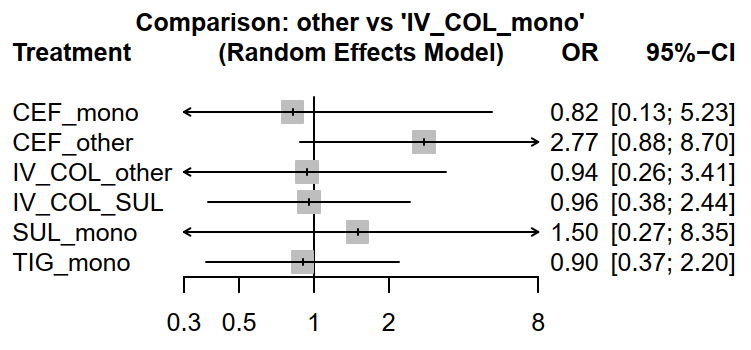


(G)


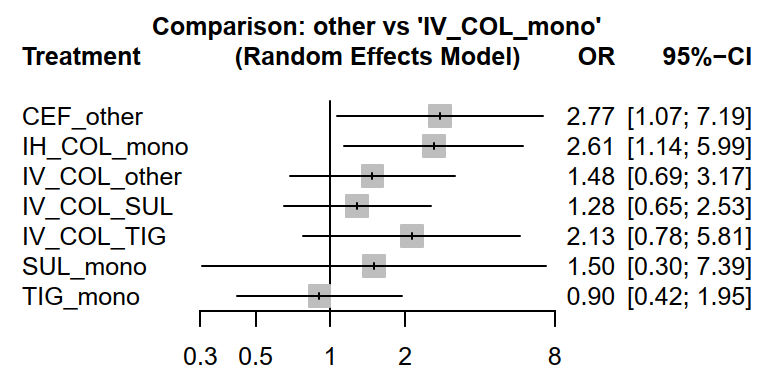


(H)

Figure S7: Forest plots from the sensitivity analysis of microbiological eradication, using the one-study removal method, are presented. Each of the 11 included studies was sequentially excluded from the analysis, and the corresponding plots are labeled from A to I.


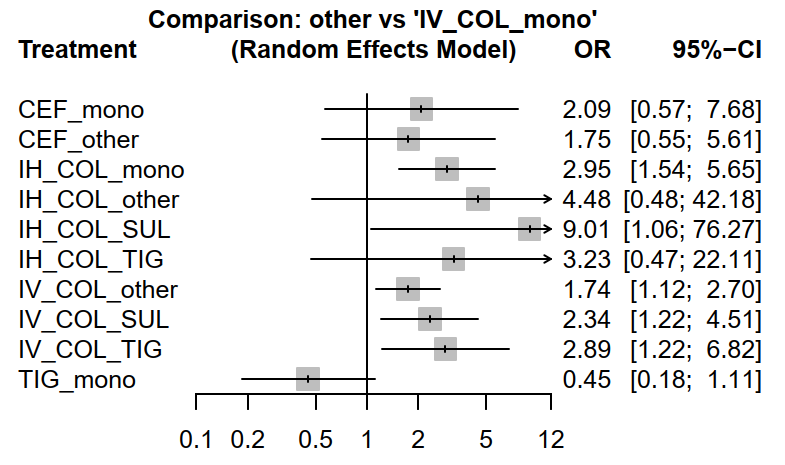


(A)


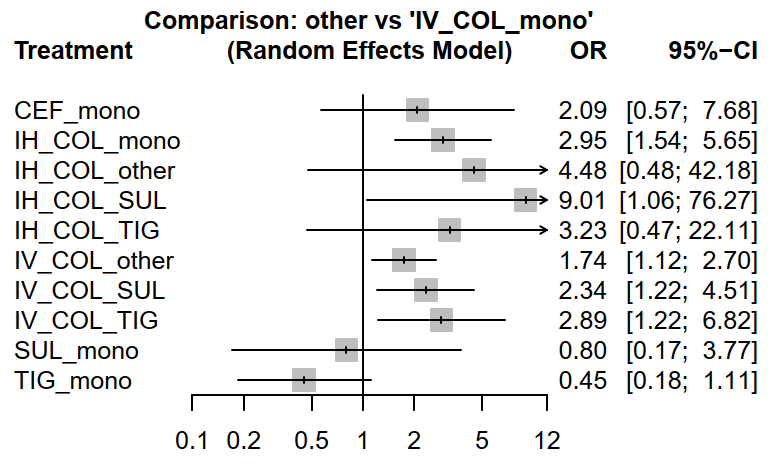


(B)


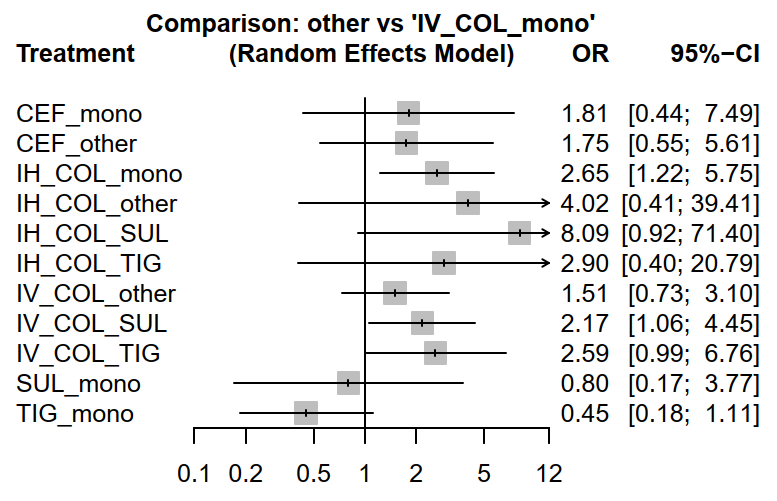


(C)


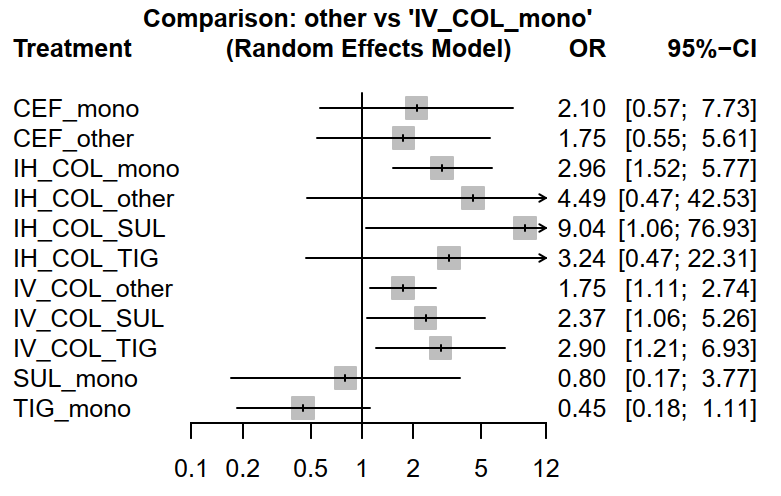


(D)


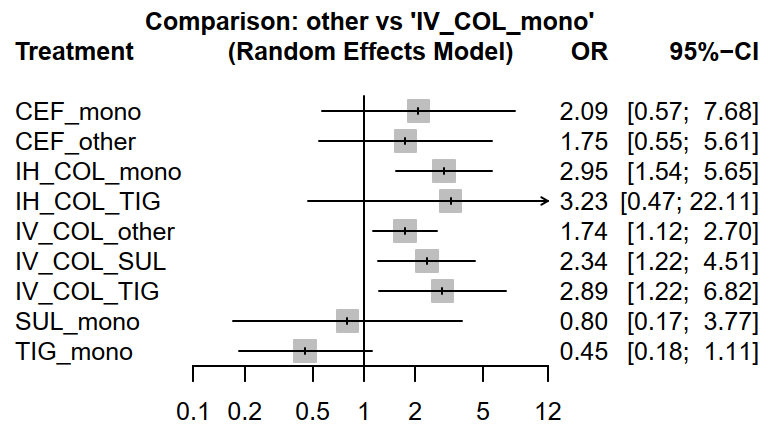


(E)


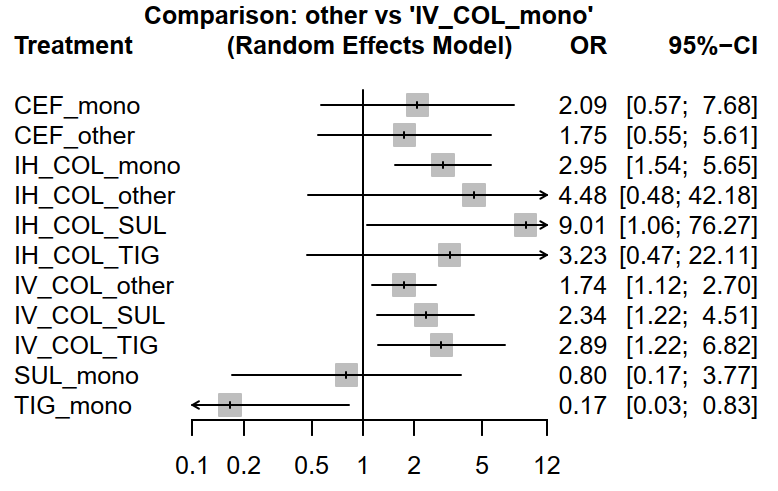


(F)


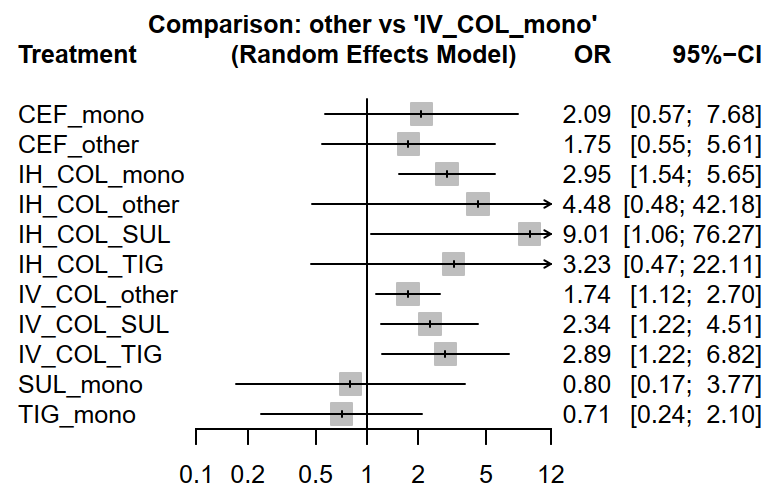


(G)


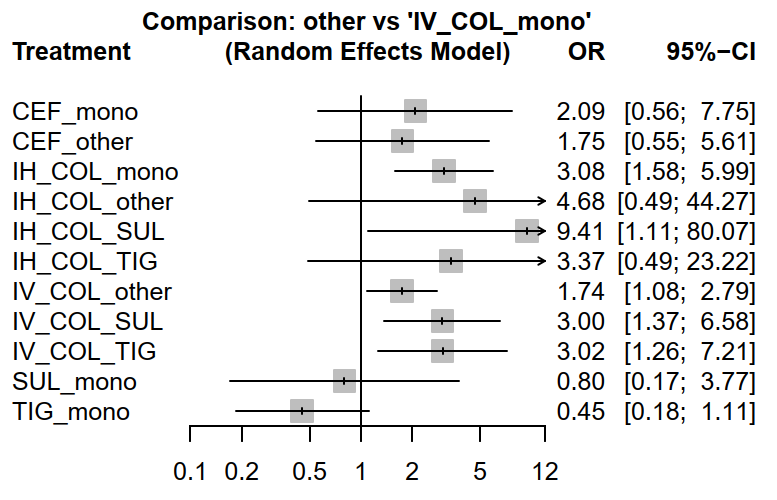


(H)


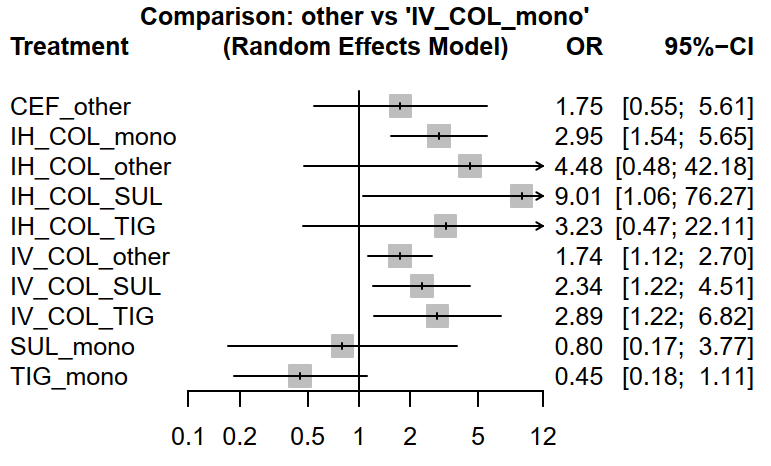


(I)

Figure S8: Forest plots from the sensitivity analysis of nephrotoxicity, using the one-study removal method, are presented. Each of the 6 included studies was sequentially excluded from the analysis, and the corresponding plots are labeled from A to E.


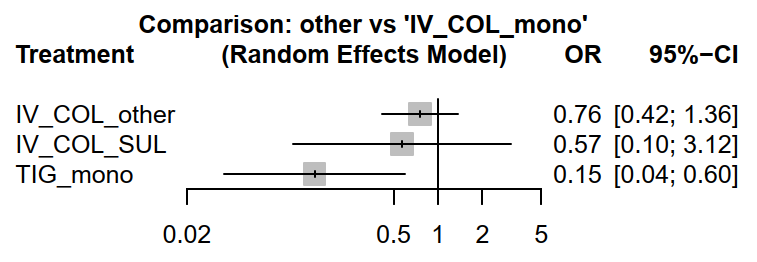


(A)


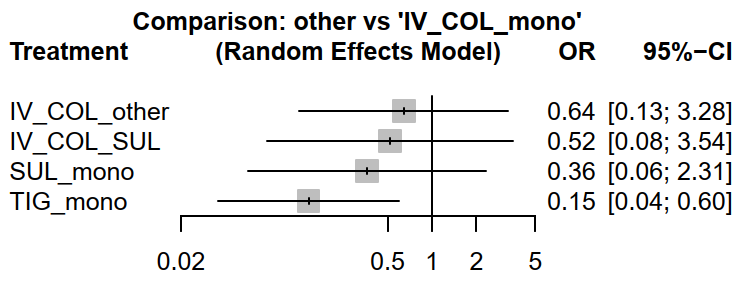


(B)


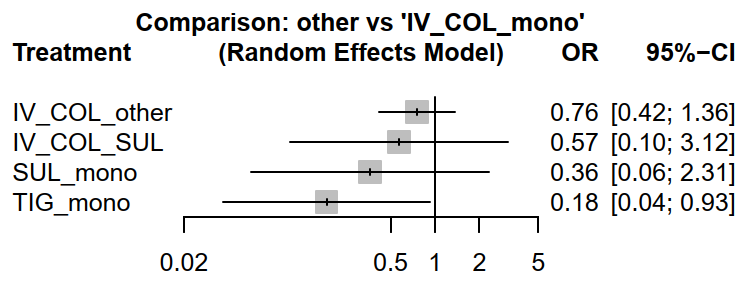


(C)


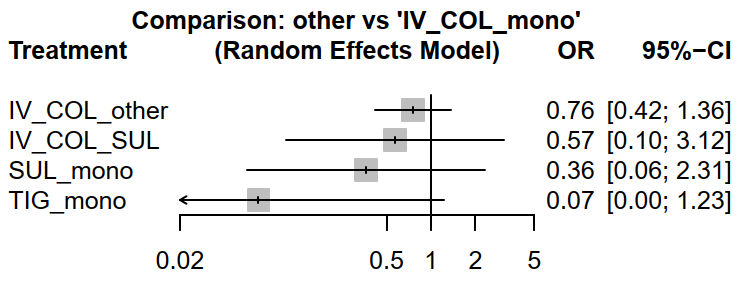


(D)


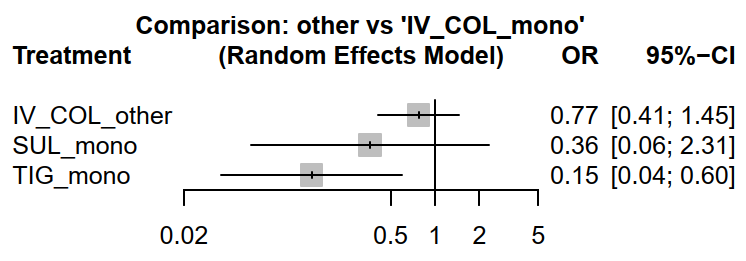


(E)

Figure S9: The funnel plot includes all paired comparisons involving the common comparator, the placebo group. Egger’s test yielded a p-value of 0.457, indicating no significant evidence of publication bias.


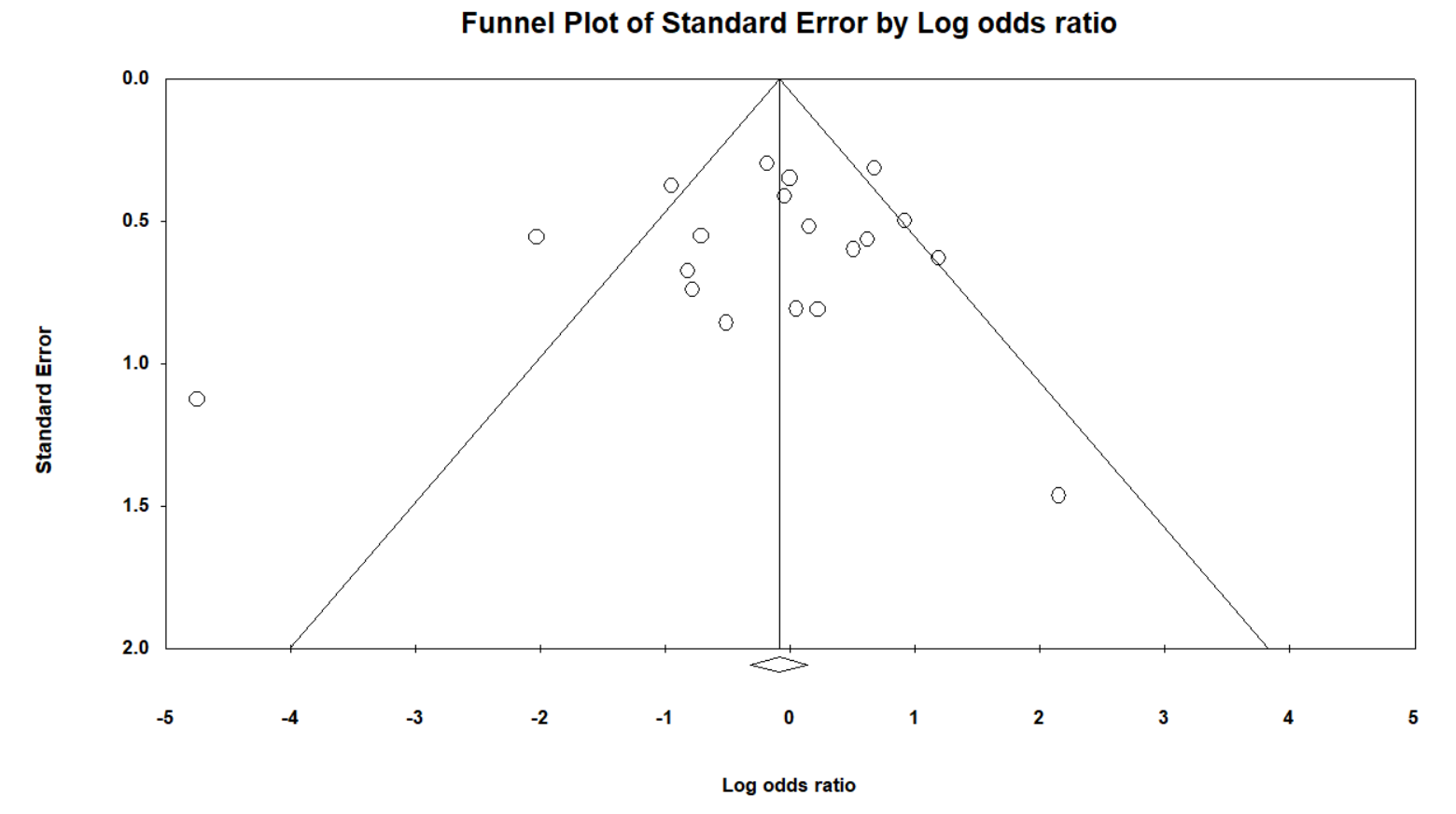


Table S1: PRISMA for network meta-analysis checklist

| **Section and Topic** | **#** | **Checklist item** | **Location** |
| --- | --- | --- | --- |
| **Title** |  |  |  |
| Title | 1 | Identify the report as a systematic review incorporating a network meta-analysis (or related form of meta-analysis). | Title |
| **Abstract** |  |  |  |
| Structured summary | 2 | Provide a structured summary including, as applicable: **Background**: main objectives / **Methods**: data sources; study eligibility criteria, participants, and interventions; study appraisal; and synthesis methods, such as network meta-analysis. / **Results**: number of studies and participants identified; summary estimates with corresponding confidence/credible intervals; treatment rankings may also be discussed. Authors may choose to summarize pairwise comparisons against a chosen treatment included in their analyses for brevity. / **Discussion/conclusions**: limitations; conclusions and  implications of findings. / **Other**: primary source of funding; systematic review registration number with registry name. | Abstract |
| **Introduction** |  |  |  |
| Rationale | 3 | Describe the rationale for the review in the context of what is already known, including mention of why a network meta-analysis has been  conducted. | Introduction |
| Objectives | 4 | Provide an explicit statement of questions being addressed, with reference to participants, interventions, comparisons, outcomes, and study design  (PICOS). | Introduction |
| **Methods** |  |  |  |
| Protocol &  registration | 5 | Indicate whether a review protocol exists and where it can be accessed; and, if available, provide registration information, including registration  number. | Materials and  Methods |
| Eligibility criteria | 6 | Specify study characteristics (e.g., PICOS, length of follow-up) and report characteristics (e.g., years considered, language, publication status) used as  criteria for eligibility, giving rationale. Clearly describe eligible treatments included in the treatment network, and note whether any have been clustered or merged into the same node (with justification). | Materials and Methods |
| Information sources | 7 | Describe all information sources (e.g., databases with dates of coverage, contact with study authors) in the search and date last searched. | Materials and  Methods |
| Search | 8 | Present full electronic search strategy for at least one database, including any limits used, such that it could be repeated. | Figure 1 |
| Study selection | 9 | State the process for selecting studies (i.e., screening, eligibility, included in systematic review, and, if applicable, included in the meta-analysis). | Figure 1 |
| Data collection | 10 | Describe method of data extraction from reports (e.g., piloted forms, independently, in duplicate) and any processes for obtaining and confirming  data. | Materials and  Methods |
| Data items | 11 | List and define all variables for which data were sought (e.g., PICOS, funding sources) and any assumptions and simplifications made. | Materials and  Methods |
| Network geometry | S1 | Describe methods used to explore the geometry of the treatment network under study and potential biases related to it. This should include how the  evidence base has been graphically summarized for presentation, and what characteristics were compiled and used to describe the evidence base to readers. | Materials and Methods |
| Risk of bias within | 12 | Describe methods used for assessing risk of bias of individual studies (including specification of whether this was done at the study or outcome level),  and how this information is to be used in any data synthesis. | Materials and  Methods |
| Summary measures | 13 | State the principal summary measures (e.g., risk ratio, difference in means). Also describe the use of additional summary measures assessed, such as  treatment rankings, as well as modified approaches used to present summary findings from meta-analyses. | Materials and  Methods |
| Planned methods of  analysis | 14 | Describe the methods of handling data and combining results of studies for each network meta-analysis. This should include, but not be limited to:  Handling of multi-arm trials; Selection of variance structure; Selection of prior distributions in Bayesian analyses; and Assessment of model fit. | Materials and  Methods |

| Assessment of  inconsistency | S2 | Describe the statistical methods used to evaluate the agreement of direct and indirect evidence in the treatment network(s) studied. Describe efforts  taken to address its presence when found. | Materials and  Methods |
| --- | --- | --- | --- |
| Risk of bias across | 15 | Specify any assessment of risk of bias that may affect the cumulative evidence. | Materials and  Methods |
| Additional analyses | 16 | Describe methods of additional analyses if done, indicating which were pre-specified. This may include, but not be limited to, the following: Sensitivity or subgroup analyses; Meta-regression analyses; Alternative formulations of the treatment network; and Use of alternative prior  distributions for Bayesian analyses (if applicable). | Materials and Methods |
| **Results** |  |  |  |
| Study selection | 17 | Give numbers of studies screened, assessed for eligibility, and included in the review, with reasons for exclusions at each stage, ideally with a flow  diagram. | Figure 1 |
| Network structure | S3 | Provide a network graph of the included studies to enable visualization of the geometry of the treatment network. | Figure 2 |
| Network geometry | S4 | Provide a brief overview of characteristics of the treatment network. This may include commentary on the abundance of trials and randomized  patients for the different interventions and pairwise comparisons in the network, gaps of evidence in the treatment network, and potential biases reflected by the network structure. | Figure 2 |
| Study  characteristics | 18 | For each study, present characteristics for which data were extracted (e.g., study size, PICOS, follow-up period) and provide the citations. | Table 1 |
| Risk of bias within | 19 | Present data on risk of bias of each study and, if available, any outcome level assessment. | Table S2 |
| Results of individual  studies | 20 | For all outcomes considered (benefits or harms), present, for each study: (1) simple summary data for each intervention group, and (2) effect  estimates and confidence intervals. Modified approaches may be needed to deal with information from larger networks. | Table 1 |
| Synthesis of results | 21 | Present results of each meta-analysis done, including confidence/credible intervals. In larger networks, authors may focus on comparisons versus a particular comparator (e.g. placebo or standard care), with full findings presented in an appendix. League tables and forest plots may be considered to summarize pairwise comparisons. If additional summary measures were explored (such as treatment rankings), these should also be presented. | Outcomes |
| Exploration for  inconsistency | S5 | Describe results from investigations of inconsistency. This may include such information as measures of model fit to compare consistency and  inconsistency models, P values from statistical tests, or summary of inconsistency estimates from different parts of the treatment network. | Table S6-9 |
| Risk of bias across | 22 | Present results of any assessment of risk of bias across studies for the evidence base being studied. | Figure S9 |
| Additional analyses | 23 | Give results of additional analyses, if done (e.g., sensitivity or subgroup analyses, meta-regression analyses, alternative network geometries studied,  alternative choice of prior distributions for Bayesian analyses, and so forth). | Figure S6-8 |
| **Discussion** |  |  |  |
| Summary of  evidence | 24 | Summarize the main findings, including the strength of evidence for each main outcome; consider their relevance to key groups. | Discussion |
| Limitations | 25 | Discuss limitations at study and outcome level (e.g., risk of bias), and at review level (e.g., incomplete retrieval of identified research, reporting bias).  Comment on the validity of the assumptions, such as transitivity and consistency. Comment on any concerns regarding network geometry (e.g., avoidance of certain comparisons). | Discussion |
| Conclusions | 26 | Provide a general interpretation of the results in the context of other evidence, and implications for future research. | Conclusions |
| **Funding** |  |  |  |
| Funding | 27 | Describe sources of funding for the systematic review and other support (e.g., supply of data); role of funders for the systematic review. This should  also include information regarding whether funding has been received from manufacturers of treatments in the network and/or whether some of the authors are content experts with professional conflicts of interest that could affect use of treatments in the network. | Funding |

PICOS, population, intervention, comparators, outcomes, study design.

Table S2. Quality assessment of included studies using the Newcastle-Ottawa Scale (NOS) and Cochrane Risk of Bias 2 (RoB 2) tool.

This table summarizes the methodological quality of included observational and randomized controlled studies. Observational studies were evaluated using the Newcastle-Ottawa Scale (NOS), covering Selection (S1–S4), Comparability (C), and Outcome (E1–E3) domains, whereas randomized controlled trials were assessed using the Cochrane Risk of Bias 2 (RoB 2) tool across five domains (randomization process, intervention adherence, missing outcome data, outcome measurement, and selective reporting.

| First Author | S^1^1 | S2 | S3 | S4 | C^2^1 | E^3^1 | E2 | E3 | Total |
| --- | --- | --- | --- | --- | --- | --- | --- | --- | --- |
| **Betrosian 2008** | ＊ | ＊ | ＊ | ＊ | ＊＊ | ＊ | ＊ | ＊ | 9 |
| **Chuang 2014** | ＊ | ＊ | ＊ | ＊ | ＊＊ |  | ＊ | ＊ | 8 |
| **Dalfino 2023** | ＊ | ＊ | ＊ | ＊ | ＊＊ | ＊ | ＊ | ＊ | 9 |
| **Deng 2022** | ＊ | ＊ | ＊ | ＊ | ＊ | ＊ | ＊ | ＊ | 8 |
| **Falcone 2022** | ＊ | ＊ | ＊ | ＊ | ＊＊ | ＊ | ＊ | ＊ | 9 |
| **Hsieh 2014** | ＊ | ＊ | ＊ | ＊ | ＊ | ＊ | ＊ | ＊ | 8 |
| **Kalin 2014** | ＊ | ＊ | ＊ | ＊ | ＊＊ | ＊ | ＊ | ＊ | 9 |
| **Khawcharoenporn**  **2014** | ＊ | ＊ | ＊ | ＊ | ＊ | ＊ | ＊ | ＊ | 8 |
| **Kim 2016** | ＊ | ＊ | ＊ | ＊ | ＊＊ | ＊ | ＊ | ＊ | 9 |
| **Kwon 2014** | ＊ | ＊ | ＊ | ＊ | ＊＊ | ＊ | ＊ | ＊ | 9 |
| **Pascale 2021** | ＊ | ＊ | ＊ | ＊ | ＊＊ |  | ＊ | ＊ | 8 |
| **Rando 2023** | ＊ | ＊ | ＊ | ＊ | ＊＊ | ＊ | ＊ | ＊ | 9 |
| **Russo 2023** | ＊ |  | ＊ | ＊ | ＊ | ＊ | ＊ | ＊ | 7 |
| **Ungthammakhun**  **2019** | ＊ | ＊ | ＊ | ＊ | ＊ | ＊ | ＊ | ＊ | 8 |
| **Yee 2024** | ＊ | ＊ | ＊ | ＊ | ＊ | ＊ | ＊ | ＊ | 8 |
| **Yilmaz 2015** | ＊ | ＊ | ＊ | ＊ | ＊＊ | ＊ | ＊ | ＊ | 9 |
| **Zheng 2019** | ＊ | ＊ | ＊ | ＊ | ＊＊ | ＊ | ＊ | ＊ | 9 |

1 The Selection part assessment.

2 The Comparability part assessment.

3 The Outcome part assessment.

| First author & Year | Randomization  process | Intervention  adherence | Missing  outcome data | Outcome  measurement | Selective  reporting | Overall  RoB |
| --- | --- | --- | --- | --- | --- | --- |
| **Bassetti 2021** | L | L | L | L | L | L |
| **Durante Mangoni 2013** | L | L | L | L | L | L |

H, high risk of bias; L, low risk of bias; S, some risk of bias.

Table S3: Pairwise comparisons and ranking of clinical success associated with different regimens used to treat MDR/XDR *Acinetobacter baumannii* pneumonia.

| **IH COL mono** | . | 1.23 [0.51, 2.96] | . | 1.63 [0.78, 3.42] | . | 1.74 [0.65, 4.66] | 3.92 [1.36, 11.35] | . |
| --- | --- | --- | --- | --- | --- | --- | --- | --- |
| 0.94 [0.27, 3.34] | **CEF + other** | . | . | . | . | . | 2.77 [1.07, 7.19] | . |
| 1.23 [0.51, 2.96] | 1.30 [0.33, 5.20] | **IV COL + TIG** | . | 1.33 [0.53, 3.38] | . | 1.42 [0.46, 4.42] | 3.20 [0.96, 10.66] | . |
| 1.74 [0.29, 10.50] | 1.85 [0.29, 11.84] | 1.42 [0.22, 9.34] | **SUL mono** | . | . | . | 1.50 [0.30, 7.39] | . |
| 1.77 [0.88, 3.56] | 1.88 [0.55, 6.36] | 1.44 [0.59, 3.54] | 1.02 [0.17, 5.95] | **IV COL + other** | 1.14 [0.36, 3.67] | 1.21 [0.56, 2.62] | 1.33 [0.56, 3.12] | . |
| 2.02 [0.52, 7.86] | 2.14 [0.40, 11.59] | 1.65 [0.38, 7.18] | 1.16 [0.14, 9.65] | 1.14 [0.36, 3.67] | **CEF mono** | . | . | . |
| 2.04 [0.92, 4.51] | 2.16 [0.67, 6.98] | 1.66 [0.63, 4.40] | 1.17 [0.21, 6.63] | 1.15 [0.56, 2.36] | 1.01 [0.26, 3.96] | **IV COL + SUL** | 1.27 [0.64, 2.52] | . |
| 2.61 [1.14, 5.99] | 2.77 [1.07, 7.19] | 2.13 [0.78 5.81] | 1.50 [0.30, 7.39] | 1.48 [0.69, 3.17] | 1.29 [0.32, 5.20] | 1.28 [0.65, 2.53] | **IV COL mono** | 1.11 [0.51, 2.38] |
| 2.88 [0.93, 8.94] | 3.06 [0.90; 10.42] | 2.35 [0.66, 8.33] | 1.66 [0.28, 9.74] | 1.63 [0.55, 4.82] | 1.43 [0.29, 7.01] | 1.42 [0.51, 3.96] | 1.11 [0.51, 2.38] | **TIG mono** |

Table S4: Pairwise comparisons and ranking of microbiological eradication associated with different regimens used to treat MDR/XDR *Acinetobacter baumannii*

pneumonia.

| **IH COL + SUL** | 2.01 [0.64, 6.28] | . | . | 2.79 [1.10, 7.06] | . | . | . | . | . | . | . |
| --- | --- | --- | --- | --- | --- | --- | --- | --- | --- | --- | --- |
| 2.01 [0.64, 6.28] | **IH COL + other** | . | . | 1.39 [0.44, 4.39] | . | . | . | . | . | . | . |
| 3.05 [0.40, 3.37] | 1.52 [0.18, 12.99] | **IH COL mono** | 1.02 [0.46, 2.26] | 0.91 [0.15, 5.58] | 0.86 [0.32, 2.33] | . | . | 1.90 [1.01, 3.55] | . | 2.88 [1.05, 7.87] | . |
| 3.12 [0.35,27.72] | 1.55 [0.16, 15.29] | 1.02 [0.46, 2.26] | **IV COL + TIG** | . | 0.85 [0.27, 2.65] | . | . | 1.86 [0.80, 4.31] | . | 2.82 [0.89, 8.92] | . |
| 2.79 [1.10, 7.06] | 1.39 [0.44, 4.39] | 0.91 [0.15, 5.58] | 0.89 [0.12, 6.45] | **IH COL + TIG** | . | . | . | . | . | . | . |
| 3.85 [0.44, 33.70] | 1.91 [0.20, 18.61] | 1.26 [0.59, 2.68] | 1.23 [0.48, 3.16] | 1.38 [0.19, 9.80] | **IV COL + SUL** | . | . | 1.44 [0.67, 3.09] | . | 2.18 [1.07, 4.47] | . |
| 4.31 [0.37, 49.64] | 2.14 [0.17, 27.10] | 1.41 [0.36, 5.46] | 1.38 [0.32, 5.98] | 1.54 [0.16,14.81] | 1.12 [0.28, 4.49] | **CEF mono** | . | 1.20 [0.35, 4.08] | . | . | . |
| 5.15 [0.45, 58.67] | 2.56 [0.20, 32.04] | 1.68 [0.44, 6.40] | 1.65 [0.39, 7.02] | 1.85 [0.19, 7.49] | 1.34 [0.35, 5.10] | 1.19 [0.21, 6.85] | **CEF + other** | . | . | 1.75 [0.55, 5.61] | . |
| 5.17 [0.62, 42.87] | 2.57 [0.28, 23.73] | 1.69 [0.95, 3.02] | 1.66 [0.74, 3.71] | 1.85 [0.28, 12.40] | 1.34 [0.70, 2.58] | 1.20 [0.35, 4.08] | 1.00 [0.29, 3.49] | **IV COL + other** | . | 1.77 [1.13, 2.77] | . |
| 11.26 [0.80,157.63] | 5.60 [0.37, 85.48] | 3.69 [0.69, 19.79] | 3.61 [0.61, 21.23] | 4.04 [0.34, 47.73] | 2.93 [0.54, 15.74] | 2.61 [0.35, 19.76] | 2.19 [0.31, 15.20] | 2.18 [0.44, 10.90] | **SUL mono** | 0.80 [0.17, 3.77] | . |
| 9.01 [1.06, 76.27] | 4.48 [0.48 42.18] | 2.95 [1.54, 5.65] | 2.89 [1.22, 6.82] | 3.23 [0.47, 22.11] | 2.34 [1.22, 4.51] | 2.09 [0.57, 7.68] | 1.75 [0.55, 5.61] | 1.74 [1.12, 2.70] | 0.80 [0.17, 3.77] | **IV COL mono** | 2.21 [0.90, 5.43] |
| 19.92 [1.96, 202.27] | 9.91 [0.88, 110.97] | 6.52 [2.15, 19.78] | 6.39 [1.84, 22.15] | 7.14 [0.85, 59.71] | 5.18 [1.70, 15.75] | 4.62 [0.95, 22.47] | 3.87 [0.89, 16.87] | 3.85 [1.42, 10.47] | 1.77 [0.30, 10.61] | 2.21 [0.90, 5.43] | **TIG mono** |

Table S5: Pairwise comparisons and ranking of nephrotoxicity associated with different regimens used to treat MDR/XDR *Acinetobacter baumannii* pneumonia.

| **TIG mono** | . | . | . | 0.15 [0.04, 0.60] |
| --- | --- | --- | --- | --- |
| 0.40 [0.04, 4.10] | **SUL mono** | . | . | 0.36 [0.06, 2.31] |
| 0.26 [0.03, 2.33] | 0.64 [0.05, 7.88] | **IV COL + SUL** | 0.81 [0.13, 4.86] | 0.52 [0.08, 3.54] |
| 0.19 [0.04, 0.89] | 0.48 [0.07, 3.35] | 0.75 [0.14, 4.06] | **IV COL + other** | 0.76 [0.42, 1.36] |
| 0.15 [0.04, 0.60] | 0.36 [0.06, 2.31] | 0.57 [0.10, 3.12] | 0.76 [0.42, 1.36] | **IV COL mono** |

Table S6: Inconsistency test results for the odds ratios (ORs) of all-cause mortality across different regimens used to treat MDR/XDR *Acinetobacter baumannii* pneumonia.

| Comparison | Studies | NMA | Direct | Indirect | Difference | 95CIL | 95CIU | p-value |
| --- | --- | --- | --- | --- | --- | --- | --- | --- |
| CEF mono: CEF + other | 0 | 2.80 | NA | 2.80 | NA | NA | NA | NA |
| CEF mono: IH COL mono | 0 | 1.59 | NA | 1.59 | NA | NA | NA | NA |
| CEF mono: IH COL + other | 0 | 1.28 | NA | 1.28 | NA | NA | NA | NA |
| CEF mono: IH COL + SUL | 0 | 1.08 | NA | 1.08 | NA | NA | NA | NA |
| CEF mono: IH COL + TIG | 0 | 1.54 | NA | 1.54 | NA | NA | NA | NA |
| CEF mono: IV COL + CAR | 0 | 3.03 | NA | 3.03 | NA | NA | NA | NA |
| CEF mono: IV COL mono | 0 | 1.39 | NA | 1.39 | NA | NA | NA | NA |
| CEF mono: IV COL + other | 1 | 1.19 | 1.19 | NA | NA | NA | NA | NA |
| CEF mono: IV COL + SUL | 0 | 1.01 | NA | 1.01 | NA | NA | NA | NA |
| CEF mono: IV COL + TIG | 0 | 0.88 | NA | 0.88 | NA | NA | NA | NA |
| CEF mono: POL + CAR | 0 | 1.57 | NA | 1.57 | NA | NA | NA | NA |
| CEF mono: SUL mono | 0 | 2.31 | NA | 2.31 | NA | NA | NA | NA |
| CEF mono: SUL + other | 0 | 1.96 | NA | 1.96 | NA | NA | NA | NA |
| CEF mono: SUL + TIG | 0 | 2.68 | NA | 2.68 | NA | NA | NA | NA |
| CEF mono: TIG mono | 0 | 1.00 | NA | 1.00 | NA | NA | NA | NA |
| CEF mono: TIG + other | 0 | 1.98 | NA | 1.98 | NA | NA | NA | NA |
| CEF + other: IH COL mono | 0 | -1.21 | NA | -1.21 | NA | NA | NA | NA |
| CEF + other: IH COL + other | 0 | -1.53 | NA | -1.53 | NA | NA | NA | NA |
| CEF + other: IH COL + SUL | 0 | -1.72 | NA | -1.72 | NA | NA | NA | NA |
| CEF + other: IH COL + TIG | 0 | -1.26 | NA | -1.26 | NA | NA | NA | NA |
| CEF + other: IV COL + CAR | 1 | 0.22 | 0.22 | NA | NA | NA | NA | NA |
| CEF + other: IV COL mono | 0 | -1.42 | NA | -1.42 | NA | NA | NA | NA |
| CEF + other: IV COL + other | 4 | -1.61 | -1.61 | NA | NA | NA | NA | NA |
| CEF + other: IV COL + SUL | 0 | -1.80 | NA | -1.80 | NA | NA | NA | NA |
| CEF + other: IV COL + TIG | 0 | -1.92 | NA | -1.92 | NA | NA | NA | NA |
| CEF + other: POL + CAR | 0 | -1.23 | NA | -1.23 | NA | NA | NA | NA |
| CEF + other: SUL mono | 0 | -0.49 | NA | -0.49 | NA | NA | NA | NA |
| CEF + other: SUL + other | 0 | -0.84 | NA | -0.84 | NA | NA | NA | NA |
| CEF + other: SUL + TIG | 0 | -0.12 | NA | -0.12 | NA | NA | NA | NA |
| CEF + other: TIG mono | 0 | -1.80 | NA | -1.80 | NA | NA | NA | NA |
| CEF + other: TIG + other | 0 | -0.83 | NA | -0.83 | NA | NA | NA | NA |
| IH COL mono: IH COL + other | 0 | -0.32 | NA | -0.32 | NA | NA | NA | NA |
| IH COL mono: IH COL + SUL | 0 | -0.51 | NA | -0.51 | NA | NA | NA | NA |
| IH COL mono: IH COL + TIG | 1 | -0.05 | -0.05 | NA | NA | NA | NA | NA |
| IH COL mono: IV COL + CAR | 0 | 1.43 | NA | 1.43 | NA | NA | NA | NA |
| IH COL mono: IV COL mono | 1 | -0.21 | -0.92 | 0.87 | -1.79 | -4.13 | 0.55 | 0.13 |
| IH COL mono: IV COL + other | 1 | -0.40 | -0.34 | -0.64 | 0.31 | -2.35 | 2.96 | 0.82 |
| IH COL mono: IV COL + SUL | 1 | -0.59 | 0.16 | -1.43 | 1.59 | -0.75 | 3.94 | 0.18 |
| IH COL mono: IV COL + TIG | 1 | -0.71 | -0.71 | NA | NA | NA | NA | NA |
| IH COL mono: POL + CAR | 0 | -0.02 | NA | -0.02 | NA | NA | NA | NA |
| IH COL mono: SUL mono | 0 | 0.72 | NA | 0.72 | NA | NA | NA | NA |
| IH COL mono: SUL + other | 0 | 0.37 | NA | 0.37 | NA | NA | NA | NA |
| IH COL mono: SUL + TIG | 0 | 1.09 | NA | 1.09 | NA | NA | NA | NA |
| IH COL mono: TIG mono | 0 | -0.59 | NA | -0.59 | NA | NA | NA | NA |
| IH COL mono: TIG + other | 0 | 0.38 | NA | 0.38 | NA | NA | NA | NA |
| IH COL + other: IH COL + SUL | 1 | -0.19 | -0.19 | NA | NA | NA | NA | NA |
| IH COL + other: IH COL + TIG | 1 | 0.27 | 0.27 | NA | NA | NA | NA | NA |
| IH COL + other: IV COL + CAR | 0 | 1.75 | NA | 1.75 | NA | NA | NA | NA |
| IH COL + other: IV COL mono | 0 | 0.11 | NA | 0.11 | NA | NA | NA | NA |
| IH COL + other: IV COL + other | 0 | -0.08 | NA | -0.08 | NA | NA | NA | NA |
| IH COL + other: IV COL + SUL | 0 | -0.27 | NA | -0.27 | NA | NA | NA | NA |
| IH COL + other: IV COL + TIG | 0 | -0.40 | NA | -0.40 | NA | NA | NA | NA |
| IH COL + other: POL + CAR | 0 | 0.29 | NA | 0.29 | NA | NA | NA | NA |
| IH COL + other: SUL mono | 0 | 1.04 | NA | 1.04 | NA | NA | NA | NA |
| IH COL + other: SUL + other | 0 | 0.69 | NA | 0.69 | NA | NA | NA | NA |
| IH COL + other: SUL + TIG | 0 | 1.40 | NA | 1.40 | NA | NA | NA | NA |
| IH COL + other: TIG mono | 0 | -0.28 | NA | -0.28 | NA | NA | NA | NA |
| IH COL + other: TIG + other | 0 | 0.70 | NA | 0.70 | NA | NA | NA | NA |
| IH COL + SUL: IH COL + TIG | 1 | 0.46 | 0.46 | NA | NA | NA | NA | NA |
| IH COL + SUL: IV COL + CAR | 0 | 1.94 | NA | 1.94 | NA | NA | NA | NA |
| IH COL + SUL: IV COL mono | 0 | 0.30 | NA | 0.30 | NA | NA | NA | NA |
| IH COL + SUL: IV COL + other | 0 | 0.11 | NA | 0.11 | NA | NA | NA | NA |
| IH COL + SUL: IV COL + SUL | 0 | -0.08 | NA | -0.08 | NA | NA | NA | NA |
| IH COL + SUL: IV COL + TIG | 0 | -0.20 | NA | -0.20 | NA | NA | NA | NA |
| IH COL + SUL: POL + CAR | 0 | 0.49 | NA | 0.49 | NA | NA | NA | NA |
| IH COL + SUL: SUL mono | 0 | 1.23 | NA | 1.23 | NA | NA | NA | NA |
| IH COL + SUL: SUL + other | 0 | 0.88 | NA | 0.88 | NA | NA | NA | NA |
| IH COL + SUL: SUL + TIG | 0 | 1.60 | NA | 1.60 | NA | NA | NA | NA |
| IH COL + SUL: TIG mono | 0 | -0.09 | NA | -0.09 | NA | NA | NA | NA |
| IH COL + SUL: TIG + other | 0 | 0.89 | NA | 0.89 | NA | NA | NA | NA |
| IH COL + TIG: IV COL + CAR | 0 | 1.48 | NA | 1.48 | NA | NA | NA | NA |
| IH COL + TIG: IV COL mono | 0 | -0.16 | NA | -0.16 | NA | NA | NA | NA |
| IH COL + TIG: IV COL + other | 0 | -0.35 | NA | -0.35 | NA | NA | NA | NA |
| IH COL + TIG: IV COL + SUL | 0 | -0.53 | NA | -0.53 | NA | NA | NA | NA |
| IH COL + TIG: IV COL + TIG | 0 | -0.66 | NA | -0.66 | NA | NA | NA | NA |
| IH COL + TIG: POL + CAR | 0 | 0.03 | NA | 0.03 | NA | NA | NA | NA |
| IH COL + TIG: SUL mono | 0 | 0.77 | NA | 0.77 | NA | NA | NA | NA |
| IH COL + TIG: SUL + other | 0 | 0.42 | NA | 0.42 | NA | NA | NA | NA |
| IH COL + TIG: SUL + TIG | 0 | 1.14 | NA | 1.14 | NA | NA | NA | NA |
| IH COL + TIG: TIG mono | 0 | -0.54 | NA | -0.54 | NA | NA | NA | NA |
| IH COL + TIG: TIG + other | 0 | 0.44 | NA | 0.44 | NA | NA | NA | NA |
| IV COL + CAR: IV COL mono | 0 | -1.64 | NA | -1.64 | NA | NA | NA | NA |
| IV COL + CAR: IV COL + other | 0 | -1.84 | NA | -1.84 | NA | NA | NA | NA |
| IV COL + CAR: IV COL + SUL | 0 | -2.02 | NA | -2.02 | NA | NA | NA | NA |
| IV COL + CAR: IV COL + TIG | 0 | -2.15 | NA | -2.15 | NA | NA | NA | NA |
| IV COL + CAR: POL + CAR | 0 | -1.46 | NA | -1.46 | NA | NA | NA | NA |
| IV COL + CAR: SUL mono | 0 | -0.71 | NA | -0.71 | NA | NA | NA | NA |
| IV COL + CAR: SUL + other | 0 | -1.06 | NA | -1.06 | NA | NA | NA | NA |
| IV COL + CAR: SUL + TIG | 0 | -0.35 | NA | -0.35 | NA | NA | NA | NA |
| IV COL + CAR: TIG mono | 0 | -2.03 | NA | -2.03 | NA | NA | NA | NA |
| IV COL + CAR: TIG + other | 0 | -1.05 | NA | -1.05 | NA | NA | NA | NA |
| IV COL + other: IV COL mono | 3 | 0.19 | -0.07 | 1.02 | -1.10 | -2.68 | 0.48 | 0.17 |
| IV COL + SUL: IV COL mono | 3 | 0.38 | 0.52 | 0.00 | 0.51 | -1.24 | 2.27 | 0.57 |
| IV COL + TIG: IV COL mono | 1 | 0.51 | -0.21 | 1.78 | -1.99 | -4.59 | 0.61 | 0.13 |
| POL + CAR: IV COL mono | 0 | -0.18 | NA | -0.18 | NA | NA | NA | NA |
| SUL mono: IV COL mono | 1 | -0.93 | -0.51 | -1.80 | 1.29 | -2.09 | 4.68 | 0.45 |
| SUL + other: IV COL mono | 1 | -0.58 | -1.95 | 0.33 | -2.28 | -6.36 | 1.81 | 0.27 |
| SUL + TIG: IV COL mono | 1 | -1.29 | -2.64 | -0.46 | -2.18 | -6.32 | 1.97 | 0.30 |
| TIG mono: IV COL mono | 3 | 0.39 | 0.48 | -1.19 | 1.67 | -1.54 | 4.88 | 0.31 |
| TIG + other: IV COL mono | 1 | -0.59 | -1.90 | 0.06 | -1.95 | -5.83 | 1.92 | 0.32 |
| IV COL + other: IV COL + SUL | 3 | -0.18 | -0.04 | -0.85 | 0.80 | -1.06 | 2.66 | 0.40 |
| IV COL + other: IV COL + TIG | 1 | -0.31 | -0.38 | -0.02 | -0.36 | -3.43 | 2.72 | 0.82 |
| IV COL + other: POL + CAR | 1 | 0.38 | 0.60 | -0.32 | 0.92 | -2.25 | 4.09 | 0.57 |
| IV COL + other: SUL mono | 1 | 1.12 | 2.04 | 0.64 | 1.40 | -2.09 | 4.89 | 0.43 |
| IV COL + other: SUL + other | 1 | 0.77 | 0.25 | 1.02 | -0.77 | -5.06 | 3.52 | 0.73 |
| IV COL + other: SUL + TIG | 1 | 1.49 | 1.35 | 1.58 | -0.23 | -4.33 | 3.86 | 0.91 |
| IV COL + other: TIG mono | 1 | -0.19 | 1.35 | -0.36 | 1.71 | -1.61 | 5.02 | 0.31 |
| IV COL + other: TIG + other | 1 | 0.79 | 0.84 | 0.73 | 0.11 | -3.53 | 3.75 | 0.95 |
| IV COL + SUL: IV COL + TIG | 1 | -0.13 | -0.87 | 0.87 | -1.75 | -4.31 | 0.82 | 0.18 |
| IV COL + SUL: POL + CAR | 0 | 0.56 | NA | 0.56 | NA | NA | NA | NA |
| IV COL + SUL: SUL mono | 0 | 1.31 | NA | 1.31 | NA | NA | NA | NA |
| IV COL + SUL: SUL + other | 0 | 0.96 | NA | 0.96 | NA | NA | NA | NA |
| IV COL + SUL: SUL + TIG | 0 | 1.67 | NA | 1.67 | NA | NA | NA | NA |
| IV COL + SUL: TIG mono | 0 | -0.01 | NA | -0.01 | NA | NA | NA | NA |
| IV COL + SUL: TIG + other | 0 | 0.97 | NA | 0.97 | NA | NA | NA | NA |
| IV COL + TIG: POL + CAR | 0 | 0.69 | NA | 0.69 | NA | NA | NA | NA |
| IV COL + TIG: SUL mono | 0 | 1.43 | NA | 1.43 | NA | NA | NA | NA |
| IV COL + TIG: SUL + other | 0 | 1.09 | NA | 1.09 | NA | NA | NA | NA |
| IV COL + TIG: SUL + TIG | 0 | 1.80 | NA | 1.80 | NA | NA | NA | NA |
| IV COL + TIG: TIG mono | 0 | 0.12 | NA | 0.12 | NA | NA | NA | NA |
| IV COL + TIG: TIG + other | 0 | 1.10 | NA | 1.10 | NA | NA | NA | NA |
| POL + CAR: SUL mono | 1 | 0.74 | 1.44 | 0.02 | 1.42 | -2.30 | 5.14 | 0.45 |
| POL + CAR: SUL + other | 1 | 0.40 | -0.35 | 0.79 | -1.14 | -5.29 | 3.01 | 0.59 |
| POL + CAR: SUL + TIG | 1 | 1.11 | 0.75 | 1.40 | -0.65 | -4.61 | 3.31 | 0.75 |
| POL + CAR: TIG mono | 1 | -0.57 | 0.75 | -1.05 | 1.80 | -1.65 | 5.25 | 0.31 |
| POL + CAR: TIG + other | 1 | 0.41 | 0.24 | 0.67 | -0.43 | -4.03 | 3.17 | 0.82 |
| SUL mono: SUL + other | 1 | -0.35 | -1.79 | 0.40 | -2.19 | -7.24 | 2.87 | 0.40 |
| SUL mono: SUL + TIG | 1 | 0.37 | -0.69 | 1.08 | -1.77 | -6.66 | 3.12 | 0.48 |
| SUL mono: TIG mono | 1 | -1.31 | -0.69 | -1.48 | 0.78 | -3.47 | 5.03 | 0.72 |
| SUL mono: TIG + other | 1 | -0.33 | -1.20 | 0.42 | -1.63 | -6.11 | 2.85 | 0.48 |
| SUL + other: SUL + TIG | 2 | 0.71 | 0.77 | -7.33 | 8.10 | -14.71 | 30.91 | 0.49 |
| SUL + other: TIG mono | 1 | -0.97 | 1.10 | -1.60 | 2.70 | -2.26 | 7.65 | 0.29 |
| SUL + other: TIG + other | 2 | 0.01 | 0.08 | -4.84 | 4.92 | -10.08 | 19.93 | 0.52 |
| SUL + TIG: TIG mono | 1 | -1.68 | 0.00 | -2.31 | 2.31 | -2.41 | 7.04 | 0.34 |
| SUL + TIG: TIG + other | 2 | -0.70 | -0.68 | -11.04 | 10.36 | -30.58 | 51.29 | 0.62 |
| TIG mono: TIG + other | 1 | 0.98 | -0.51 | 1.60 | -2.11 | -6.34 | 2.12 | 0.33 |

Table S7: Inconsistency test results for the odds ratios (ORs) of clinical success across different regimens used to treat MDR/XDR *Acinetobacter baumannii* pneumonia.

| Comparison | Studies | NMA | Direct | Indirect | Difference | 95CIL | 95CIU | p-value |
| --- | --- | --- | --- | --- | --- | --- | --- | --- |
| CEF mono: CEF + other | 0 | -0.76 | NA | -0.76 | NA | NA | NA | NA |
| CEF mono: IH COL mono | 0 | -0.70 | NA | -0.70 | NA | NA | NA | NA |
| CEF mono: IV COL mono | 0 | 0.26 | NA | 0.26 | NA | NA | NA | NA |
| CEF mono: IV COL + other | 1 | -0.13 | -0.13 | NA | NA | NA | NA | NA |
| CEF mono: IV COL + SUL | 0 | 0.01 | NA | 0.01 | NA | NA | NA | NA |
| CEF mono: IV COL + TIG | 0 | -0.50 | NA | -0.50 | NA | NA | NA | NA |
| CEF mono: SUL mono | 0 | -0.15 | NA | -0.15 | NA | NA | NA | NA |
| CEF mono: TIG mono | 0 | 0.36 | NA | 0.36 | NA | NA | NA | NA |
| CEF + other: IH COL mono | 0 | 0.06 | NA | 0.06 | NA | NA | NA | NA |
| CEF + other: IV COL mono | 1 | 1.02 | 1.02 | NA | NA | NA | NA | NA |
| CEF + other: IV COL + other | 0 | 0.63 | NA | 0.63 | NA | NA | NA | NA |
| CEF + other: IV COL + SUL | 0 | 0.77 | NA | 0.77 | NA | NA | NA | NA |
| CEF + other: IV COL + TIG | 0 | 0.26 | NA | 0.26 | NA | NA | NA | NA |
| CEF + other: SUL mono | 0 | 0.61 | NA | 0.61 | NA | NA | NA | NA |
| CEF + other: TIG mono | 0 | 1.12 | NA | 1.12 | NA | NA | NA | NA |
| IH COL mono: IV COL mono | 1 | 0.96 | 1.37 | 0.32 | 1.05 | -0.65 | 2.76 | 0.23 |
| IH COL mono: IV COL + other | 1 | 0.57 | 0.49 | 1.24 | -0.75 | -3.05 | 1.55 | 0.52 |
| IH COL mono: IV COL + SUL | 1 | 0.71 | 0.56 | 1.00 | -0.44 | -2.11 | 1.22 | 0.60 |
| IH COL mono: IV COL + TIG | 1 | 0.20 | 0.20 | NA | NA | NA | NA | NA |
| IH COL mono: SUL mono | 0 | 0.55 | NA | 0.55 | NA | NA | NA | NA |
| IH COL mono: TIG mono | 0 | 1.06 | NA | 1.06 | NA | NA | NA | NA |
| IV COL + other: IV COL mono | 2 | 0.39 | 0.28 | 0.80 | -0.52 | -2.40 | 1.37 | 0.59 |
| IV COL + SUL: IV COL mono | 3 | 0.25 | 0.24 | 7.01 | -6.77 | -27.52 | 13.99 | 0.52 |
| IV COL + TIG: IV COL mono | 1 | 0.75 | 1.16 | -0.19 | 1.35 | -0.84 | 3.54 | 0.23 |
| SUL mono: IV COL mono | 1 | 0.41 | 0.41 | NA | NA | NA | NA | NA |
| TIG mono: IV COL mono | 2 | -0.10 | -0.10 | NA | NA | NA | NA | NA |
| IV COL + other: IV COL + SUL | 2 | 0.14 | 0.19 | -0.17 | 0.36 | -1.70 | 2.42 | 0.73 |
| IV COL + other: IV COL + TIG | 1 | -0.37 | -0.29 | -1.47 | 1.19 | -2.45 | 4.83 | 0.52 |
| IV COL + other: SUL mono | 0 | -0.02 | NA | -0.02 | NA | NA | NA | NA |
| IV COL + other: TIG mono | 0 | 0.49 | NA | 0.49 | NA | NA | NA | NA |
| IV COL + SUL: IV COL + TIG | 1 | -0.51 | -0.35 | -0.94 | 0.59 | -1.63 | 2.81 | 0.60 |
| IV COL + SUL: SUL mono | 0 | -0.16 | NA | -0.16 | NA | NA | NA | NA |
| IV COL + SUL: TIG mono | 0 | 0.35 | NA | 0.35 | NA | NA | NA | NA |
| IV COL + TIG: SUL mono | 0 | 0.35 | NA | 0.35 | NA | NA | NA | NA |
| IV COL + TIG: TIG mono | 0 | 0.86 | NA | 0.86 | NA | NA | NA | NA |
| SUL mono: TIG mono | 0 | 0.51 | NA | 0.51 | NA | NA | NA | NA |

Table S8: Inconsistency test results for the odds ratios (ORs) of microbiological eradication across different regimens used to treat MDR/XDR *Acinetobacter baumannii* pneumonia.

| Comparison | Studies | NMA | Direct | Indirect | Difference | 95CIL | 95CIU | p-value |
| --- | --- | --- | --- | --- | --- | --- | --- | --- |
| CEF mono: CEF + other | 0 | 0.18 | NA | 0.18 | NA | NA | NA | NA |
| CEF mono: IH COL mono | 0 | -0.34 | NA | -0.34 | NA | NA | NA | NA |
| CEF mono: IH COL + other | 0 | -0.76 | NA | -0.76 | NA | NA | NA | NA |
| CEF mono: IH COL + SUL | 0 | -1.46 | NA | -1.46 | NA | NA | NA | NA |
| CEF mono: IH COL + TIG | 0 | -0.43 | NA | -0.43 | NA | NA | NA | NA |
| CEF mono: IV COL mono | 0 | 0.74 | NA | 0.74 | NA | NA | NA | NA |
| CEF mono: IV COL + other | 1 | 0.18 | 0.18 | NA | NA | NA | NA | NA |
| CEF mono: IV COL + SUL | 0 | -0.11 | NA | -0.11 | NA | NA | NA | NA |
| CEF mono: IV COL + TIG | 0 | -0.32 | NA | -0.32 | NA | NA | NA | NA |
| CEF mono: SUL mono | 0 | 0.96 | NA | 0.96 | NA | NA | NA | NA |
| CEF mono: TIG mono | 0 | 1.53 | NA | 1.53 | NA | NA | NA | NA |
| CEF + other: IH COL mono | 0 | -0.52 | NA | -0.52 | NA | NA | NA | NA |
| CEF + other: IH COL + other | 0 | -0.94 | NA | -0.94 | NA | NA | NA | NA |
| CEF + other: IH COL + SUL | 0 | -1.64 | NA | -1.64 | NA | NA | NA | NA |
| CEF + other: IH COL + TIG | 0 | -0.61 | NA | -0.61 | NA | NA | NA | NA |
| CEF + other: IV COL mono | 1 | 0.56 | 0.56 | NA | NA | NA | NA | NA |
| CEF + other: IV COL + other | 0 | 0.00 | NA | 0.00 | NA | NA | NA | NA |
| CEF + other: IV COL + SUL | 0 | -0.29 | NA | -0.29 | NA | NA | NA | NA |
| CEF + other: IV COL + TIG | 0 | -0.50 | NA | -0.50 | NA | NA | NA | NA |
| CEF + other: SUL mono | 0 | 0.78 | NA | 0.78 | NA | NA | NA | NA |
| CEF + other: TIG mono | 0 | 1.35 | NA | 1.35 | NA | NA | NA | NA |
| IH COL mono: IH COL + other | 0 | -0.42 | NA | -0.42 | NA | NA | NA | NA |
| IH COL mono: IH COL + SUL | 0 | -1.12 | NA | -1.12 | NA | NA | NA | NA |
| IH COL mono: IH COL + TIG | 1 | -0.09 | -0.09 | NA | NA | NA | NA | NA |
| IH COL mono: IV COL mono | 1 | 1.08 | 1.06 | 1.10 | -0.04 | -1.36 | 1.28 | 0.95 |
| IH COL mono: IV COL + other | 1 | 0.53 | 0.64 | -0.12 | 0.76 | -0.86 | 2.38 | 0.36 |
| IH COL mono: IV COL + SUL | 1 | 0.23 | -0.15 | 0.75 | -0.89 | -2.42 | 0.64 | 0.25 |
| IH COL mono: IV COL + TIG | 1 | 0.02 | 0.02 | NA | NA | NA | NA | NA |
| IH COL mono: SUL mono | 0 | 1.30 | NA | 1.30 | NA | NA | NA | NA |
| IH COL mono: TIG mono | 0 | 1.88 | NA | 1.88 | NA | NA | NA | NA |
| IH COL + other: IH COL + SUL | 1 | -0.70 | -0.70 | NA | NA | NA | NA | NA |
| IH COL + other: IH COL + TIG | 1 | 0.33 | 0.33 | NA | NA | NA | NA | NA |
| IH COL + other: IV COL mono | 0 | 1.50 | NA | 1.50 | NA | NA | NA | NA |
| IH COL + other: IV COL + other | 0 | 0.94 | NA | 0.94 | NA | NA | NA | NA |
| IH COL + other: IV COL + SUL | 0 | 0.65 | NA | 0.65 | NA | NA | NA | NA |
| IH COL + other: IV COL + TIG | 0 | 0.44 | NA | 0.44 | NA | NA | NA | NA |
| IH COL + other: SUL mono | 0 | 1.72 | NA | 1.72 | NA | NA | NA | NA |
| IH COL + other: TIG mono | 0 | 2.29 | NA | 2.29 | NA | NA | NA | NA |
| IH COL + SUL: IH COL + TIG | 1 | 1.03 | 1.03 | NA | NA | NA | NA | NA |
| IH COL + SUL: IV COL mono | 0 | 2.20 | NA | 2.20 | NA | NA | NA | NA |
| IH COL + SUL: IV COL + other | 0 | 1.64 | NA | 1.64 | NA | NA | NA | NA |
| IH COL + SUL: IV COL + SUL | 0 | 1.35 | NA | 1.35 | NA | NA | NA | NA |
| IH COL + SUL: IV COL + TIG | 0 | 1.14 | NA | 1.14 | NA | NA | NA | NA |
| IH COL + SUL: SUL mono | 0 | 2.42 | NA | 2.42 | NA | NA | NA | NA |
| IH COL + SUL: TIG mono | 0 | 2.99 | NA | 2.99 | NA | NA | NA | NA |
| IH COL + TIG: IV COL mono | 0 | 1.17 | NA | 1.17 | NA | NA | NA | NA |
| IH COL + TIG: IV COL + other | 0 | 0.62 | NA | 0.62 | NA | NA | NA | NA |
| IH COL + TIG: IV COL + SUL | 0 | 0.32 | NA | 0.32 | NA | NA | NA | NA |
| IH COL + TIG: IV COL + TIG | 0 | 0.11 | NA | 0.11 | NA | NA | NA | NA |
| IH COL + TIG: SUL mono | 0 | 1.40 | NA | 1.40 | NA | NA | NA | NA |
| IH COL + TIG: TIG mono | 0 | 1.97 | NA | 1.97 | NA | NA | NA | NA |
| IV COL + other: IV COL mono | 3 | 0.56 | 0.57 | 0.30 | 0.27 | -1.64 | 2.19 | 0.78 |
| IV COL + SUL: IV COL mono | 3 | 0.85 | 0.78 | 1.20 | -0.42 | -2.18 | 1.33 | 0.64 |
| IV COL + TIG: IV COL mono | 1 | 1.06 | 1.04 | 1.09 | -0.05 | -1.79 | 1.68 | 0.95 |
| SUL mono: IV COL mono | 1 | -0.22 | -0.22 | NA | NA | NA | NA | NA |
| TIG mono: IV COL mono | 2 | -0.79 | -0.79 | NA | NA | NA | NA | NA |
| IV COL + other: IV COL + SUL | 2 | -0.30 | -0.36 | -0.12 | -0.24 | -1.70 | 1.22 | 0.75 |
| IV COL + other: IV COL + TIG | 1 | -0.50 | -0.62 | 0.75 | -1.37 | -4.30 | 1.56 | 0.36 |
| IV COL + other: SUL mono | 0 | 0.78 | NA | 0.78 | NA | NA | NA | NA |
| IV COL + other: TIG mono | 0 | 1.35 | NA | 1.35 | NA | NA | NA | NA |
| IV COL + SUL: IV COL + TIG | 1 | -0.21 | 0.17 | -1.01 | 1.18 | -0.84 | 3.20 | 0.25 |
| IV COL + SUL: SUL mono | 0 | 1.07 | NA | 1.07 | NA | NA | NA | NA |
| IV COL + SUL: TIG mono | 0 | 1.65 | NA | 1.65 | NA | NA | NA | NA |
| IV COL + TIG: SUL mono | 0 | 1.28 | NA | 1.28 | NA | NA | NA | NA |
| IV COL + TIG: TIG mono | 0 | 1.85 | NA | 1.85 | NA | NA | NA | NA |
| SUL mono: TIG mono | 0 | 0.57 | NA | 0.57 | NA | NA | NA | NA |

Table S9: Inconsistency test results for the odds ratios (ORs) of nephrotoxicity across different regimens used to treat MDR/XDR *Acinetobacter baumannii* pneumonia.

| Comparison | Studies | NMA | Direct | Indirect | Difference | 95CIL | 95CIU | p-value |
| --- | --- | --- | --- | --- | --- | --- | --- | --- |
| IV COL + other: IV COL mono | 2 | -0.28 | -0.28 | NA | NA | NA | NA | NA |
| IV COL + SUL: IV COL mono | 1 | -0.56 | -0.66 | -0.22 | -0.44 | -4.58 | 3.70 | 0.84 |
| SUL mono: IV COL mono | 1 | -1.01 | -1.01 | NA | NA | NA | NA | NA |
| TIG mono: IV COL mono | 2 | -1.92 | -1.92 | NA | NA | NA | NA | NA |
| IV COL + other: IV COL + SUL | 1 | 0.28 | 0.22 | 0.76 | -0.54 | -5.70 | 4.61 | 0.84 |
| IV COL + other: SUL mono | 0 | 0.73 | NA | 0.73 | NA | NA | NA | NA |
| IV COL + other: TIG mono | 0 | 1.64 | NA | 1.64 | NA | NA | NA | NA |
| IV COL + SUL: SUL mono | 0 | 0.45 | NA | 0.45 | NA | NA | NA | NA |
| IV COL + SUL: TIG mono | 0 | 1.36 | NA | 1.36 | NA | NA | NA | NA |
| SUL mono: TIG mono | 0 | 0.91 | NA | 0.91 | NA | NA | NA | NA |
